# Kettlebell training in clinical practice: a scoping review

**DOI:** 10.1186/s13102-019-0130-z

**Published:** 2019-09-03

**Authors:** Neil J. Meigh, Justin W. L. Keogh, Ben Schram, Wayne A. Hing

**Affiliations:** 1 Faculty of Health Sciences and Medicine, Bond University, Institute of Health & Sport, Gold Coast, Queensland 4226 Australia; 2 Sports Performance Research Centre New Zealand, AUT University, Auckland, New Zealand; 3 Kasturba Medical College, Manipal Academy of Higher Education Mangalore, Manipal, Karnataka India

**Keywords:** Scoping review, Kettlebell, Physiotherapy, Exercise

## Abstract

**Background:**

A scoping review of scientific literature on the effects of kettlebell training. There are no authoritative guidelines or recommendations for using kettlebells within a primary care setting. Our review objectives were to identify the extent, range and nature of the available evidence, to report on the types of evidence currently available to inform clinical practice, to synthesise key concepts, and identify gaps in the research knowledge base.

**Methods:**

Following the PRISMA-ScR Checklist, we conducted a search of 10 electronic databases from inception to 1 February 2019. There were no exclusions in searching for publications. A single reviewer screened the literature and abstracted data from relevant publications. Articles were grouped and charted by concepts and themes relevant to primary care, and narratively synthesised. Effect sizes from longitudinal studies were identified or calculated, and randomised controlled trials assessed for methodological quality.

**Results:**

Eight hundred and twenty-nine records were identified to 1 February 2019. Four hundred and ninety-six were screened and 170 assessed for eligibility. Ninety-nine publications met the inclusion criteria. Effect sizes were typically trivial to small. One trial used a pragmatic hardstyle training program among healthy college-age participants. Two trials reported the effects of kettlebell training in clinical conditions. Thirty-three studies explicitly used ‘hardstyle’ techniques and 4 investigated kettlebell sport. Also included were 6 reviews, 22 clinical/expert opinions and 3 case reports of injury. Two reviewers independently evaluated studies using a modified Downs & Black checklist.

**Conclusions:**

A small number of longitudinal studies, which are largely underpowered and of low methodological quality, provide the evidence-informed therapist with little guidance to inform the therapeutic prescription of kettlebells within primary care. Confidence in reported effects is low to very low. The strength of recommendation for kettlebell training improving measures of physical function is weak, based on the current body of literature. Further research on reported effects is warranted, with inclusion of clinical populations and investigations of musculoskeletal conditions common to primary care. There is a need for an externally valid, standardised approach to the training and testing of kettlebell interventions, which better informs the therapeutic use of kettlebells in primary care.

## Background

### History

The kettlebell is a round-shaped steel or cast iron weight, commonly described as resembling a cannonball with a handle [1]. In Russia, kettlebells are a matter of pride and a symbol of strength, with a colourful history throughout the twentieth Century from circus strong men to the Red Army. Use of kettlebells as measures of weight dates back to Russia in the 1700s [2] and the word *girya* (kettlebell) first appears in a Russian dictionary in 1704 [3], with excavations in Poland pre-dating early kettlebells to the seventeenth century [4].

Kettlebell sport, also referred to as Girevoy Sport originated in Eastern Europe in 1948 [5]. The International Union of Kettlebell Lifting World Championship held in October 2018 attracted more than 500 competitors from 32 countries, testament to its popularity and growth. Kettlebell sport uses competition kettlebells of standardised dimensions made of steel, most commonly available from 8 kg to 32 kg in 2-4 kg increments. Kettlebell sport techniques are the jerk and snatch in different timed events.

Kettlebells described as ‘traditional’ in shape are typically made from cast iron, with dimensions increasing with weight. Kettlebells are now widely available in an array of construction materials, from 2 kg to 92 kg. With increasing popularity has come diversity in use and adaptation of common exercises, however only a limited number of styles are widely recognised: Sport, hardstyle, juggling, and a small number of techniques associated with CrossFit.

The popularity of kettlebells outside of Eastern Europe and kettlebell sport can be largely attributed to Russian émigrés former World Champion Valery Fedorenko, and former Soviet Special Forces physical training instructor and Master of Sport, Pavel Tsatsouline. Fedorenko founded the American Kettlebell Club and Tsatsouline the hardstyle Russian Kettlebell Certification (RKC), which commenced training in 2001. Pavel has been widely credited with introducing kettlebells to the West [6] following a publication in the December 1998, Vol. 6, No. 3 Issue of MILO A Journal For Serious Strength Training Athletes. That was followed by Power to the People [7] which outlines many of the training principles used in Enter the Kettlebell [3], and remains the foundation of hardstyle kettlebell training courses worldwide. Enter the Kettlebell has been the most widely cited text in academic publications where a hardstyle technique has been used. The six fundamental hardstyle techniques are the Swing, Clean, Press, Squat, Snatch and Turkish get-up (TGU). Academic investigation of hardstyle training represents around 50% of publications (refer to Results: *report characteristics*), with the two-handed kettlebell swing investigated most frequently. Neither kettlebell sport nor hardstyle are limited to only the techniques listed.

A third person of note is former Master RKC, Kenneth Jay. A small unpublished Bachelor of Science study completed at the University of Copenhagen [8] investigated the VO_2_ and lactate effects from two weeks of dedicated hardstyle kettlebell snatch training in a group of well-conditioned, kettlebell-trained college-age males. Jay’s training protocols later described in Viking Warrior Conditioning [9] and those from Enter the Kettlebell represent the majority of study formats used to date.

#### Conceptual and contextual background

Exercise prescription is an integral part of Physiotherapy practice [10]. Prescription of exercise as medicine for a broad range of chronic diseases and for relieving pain and improving musculoskeletal function have been described [11, 12] with many at least as effective as drug therapy [13]. The mechanisms of mechanotherapy in clinical practice have been reported [14, 15], with an understanding of mechanobiology of musculoskeletal tissues critical to primary care [16]. Therapists commonly seek to increase tissue capacity and build physical and psychological resilience in their patients, from the young injured athlete to the elderly and frail.

Evidence-based Physiotherapy is an area of study, research, and practice in which clinical decisions are based on the best available evidence, integrating professional practice and expertise with ethical principles [17]. Where high quality clinical research does not exist, good practice must be informed by knowledge derived from other sources of information. When relevant and reliable data is not available, clinicians still need to make decisions based upon the best available information [18].

In elite sport, there is a constant need to increase strength, power and endurance, and the kettlebell has become a part of that effort [19]. Kettlebells have been used in strength and conditioning research and injury prevention programs for mixed martial arts [20], handball [21], shot put [22], sprinting [23] and soccer [24]. In clinical practice, kettlebells have been included in programs for lower limb amputees [25], metabolic syndrome in women [26], early treatment of breast cancer [27], for osteoporosis and fall and fracture prevention [28], home-based Physiotherapy with older adults showing signs of frailty and following hip fracture [29], for healthcare workers [30] and in programs for improving health-related physical fitness [31].

Military and law enforcement agencies train with kettlebells, reporting improvements in field performance [32]. Kettlebells have been recommended as part of the Royal Air Force aircrew conditioning programme [33] and for simulated military task performance [34]. The kettlebell deadlift has been recommended by the North Atlantic Treaty Organization to be used alongside the Ranger test, which is a loaded step test, deemed to have excellent content validity and high inter-rater reliability in relation to five common physically demanding military work tasks for soldiers [35].

Kettlebells have also been used to modify other common training protocols [36–38], and as a novel method of providing valgus stress with good reliability, during ultrasound examination of the ulnar collateral ligament of the elbow [39]. University studies have investigated kettlebell training, including analysis of the TGU [40], for improving dynamic knee stability and performance in female netball players [41], in anterior cruciate ligament (ACL) injury prevention among female athletes [42], and for reducing work related musculoskeletal disorders of the low back [43].

Whilst kettlebells have been adopted by popular fitness programs such as CrossFit, the use of kettlebells remains a relatively niche sport and knowing how to use a kettlebell is perhaps not as intuitive as the more popular barbells, dumbbells and machine weights. In spite of this, kettlebells have been recommended for their ease of teaching, cost effectiveness and being less intimidating to use [44]. Kettlebells have already been integrated into clinical practice but does the current body of evidence support their use for therapeutic purposes, and how does the evidence help inform clinical decision making?

The aim of this review is to identify what is known about the effect of kettlebell training from published academic research, with the objective to systematically evaluate and critically appraise the literature and highlight areas for further investigation.

### Kettlebell swing descriptors

The ‘hip hinge’ is associated with a deadlift movement pattern and a hardstyle kettlebell swing. This has also been described as a “Russian swing”, or a swing to chest height. It can be performed with one or two hands holding the bell. The two-handed overhead swing is associated with a ‘squatting’ motion of the lower limbs, also described as an “American swing” and most commonly linked with CrossFit. The ‘double-knee-bend’ pattern is associated with kettlebell sport.

## Methods

A scoping review was conducted to synthesise current evidence of kettlebell training as it applies to therapists working in primary care, where movement and loading are used clinically for therapeutic purposes. As an evolving field of research, the scoping review was chosen to provide an overview of kettlebell training, to identify key concepts, knowledge gaps, and types of evidence currently available.

### Research question

What evidence is available to guide therapists using kettlebells within a clinical therapeutic framework?

### Protocol

This scoping review was conducted by a single researcher (NM) using the PRISMA Extension for Scoping Reviews (PRISMA-ScR): Checklist and Explanation [45]. A priori protocol was not developed.

### Study design

The scoping methodology proposed by Arksey and O’Malley [46] was used to map the concepts and types of science-based evidence that exists on kettlebell training. The methodology was informed by later recommendations [47] and guided by the Joanna Briggs Institute framework [48, 49]. This framework includes the following steps: 1) Identify the research question by clarifying and linking the purpose and research question, 2) identify relevant studies by balancing feasibility with breadth and comprehensiveness, 3) select studies using an iterative team approach to study selection and data extraction, 4) chart the data incorporating numerical summary and qualitative thematic analysis, 5) collate, summarize and report the results, including the implications for policy, practice or research [50].

### Information sources and literature search

A search was conducted, assisted by a health sciences librarian, on 10 electronic databases (CINAHL, Cochrane Library, Embase, Medline, PEDro, ProQuest, PubMed, SportDISCUS, Web of Science, Google Scholar) from inception to 1 February 2019, using search terms “kettlebell”, “kettle bell”, “kettlebells”, “kettle bells” in the Title or Abstract. The search strategy was not limited by study design, publication type, or language. Duplicate records were removed in EndNote. Backward reference searching was performed, and additional studies were identified by consultation with subject matter experts.

### Eligibility criteria

The eligibility criteria were defined by the *Population* (therapists in primary care), *Concept* (prescription of kettlebells for therapeutic purposes) and *Context* (evidence-based practice: research evidence and clinical expertise). All types of study design and reviews were included where kettlebells were the primary modality of investigation. Any population, intervention, comparator, outcome, and setting were included, together with theses and unpublished material from academic settings. Articles/publications were excluded if, a) they were unrelated to kettlebell training (e.g. gave historical context only), b) were not specific to kettlebell training (e.g. interventions involving kettlebells and other equipment where the outcome(s) could not be attributed to the kettlebell), c) were unavailable in full text, or d) were studies conducted on Eastern European Military populations. The absence of standardised reporting guidelines (as recommended by the Enhancing the QUAlity and Transparency Of health Research network), and style of reporting from countries of the former Soviet Union, were deemed incompatible for synthesis. The following were also excluded from our review: books, patents, fitness articles, web pages, blogs and opinion pieces from non-clinical or non-academic/clinical authors. Resource limitations precluded the translation of articles not published in English. One exception was a clinical trial of hardstyle kettlebell training for people with Parkinson’s disease, published in Portuguese with an English abstract; this was deemed to be specifically relevant to the population, concept and context of the review and included but not translated. All levels of evidence [51] were considered.

### Data abstraction and data items

A standardised data abstraction form was not utilised. A single reviewer (NM) independently screened titles and abstracts for relevance and obtained full text articles of publications potentially relevant. As the scope and nature of the available evidence was not known in advance, the development of categories and grouping for mapping purposes was developed iteratively as the data was extracted and tabulated. Effect sizes were extracted where given, or calculated if enough data had been provided. Cohen’s δ or standardised mean difference (SMD) were used and magnitude of effect compared based on participant’s resistance training status: untrained, recreationally trained or highly trained [52].

### Methodological quality appraisal

With the primary intent to inform clinical practice, the authors chose to critically appraise the randomised controlled trials using a modified Downs and Black quality checklist [53]. This scoring system is based on a checklist of 27 questions and has been found to be valid and reliable for critically evaluating experimental and nonexperimental studies. The checklist included 4 categories for evaluation: reporting, external validity, internal validity/bias, and internal validity/confounding [54]. Studies were appraised by a second independent reviewer. Discrepancies were resolved by discussion and agreement reached. Quality of evidence and strength of recommendation was based upon the GRADE approach [55, 56].

### Synthesis

Data were narratively synthesised by author-defined category: (1) acute profiling, (2) athletic performance, (3) health-related physical fitness, (4) injury & rehabilitation, (5) expert/clinical option and (6) Review, with key characteristics and findings discussed. Publications were grouped by nature of the study (acute vs longitudinal) and measures/outcomes. Acute profiling studies were further categorised by outcome: ‘sEMG’, ‘motion analysis’, ‘hormonal response’, ‘cardiometabolic’, ‘mechanical demand’ or ‘performance’. Experiments and trials were mapped based on the population profile (age, gender, training history, kettlebell experience), types of exercise(s) used, style (hardstyle, sport or ‘other’), training format (work-to-rest ratio, frequency, duration, intensity/load), measurements (sEMG, motion analysis, ground reaction force, HR, RPE, VO_2_), outcomes, and study design.

## Results

**Fig. 1:**
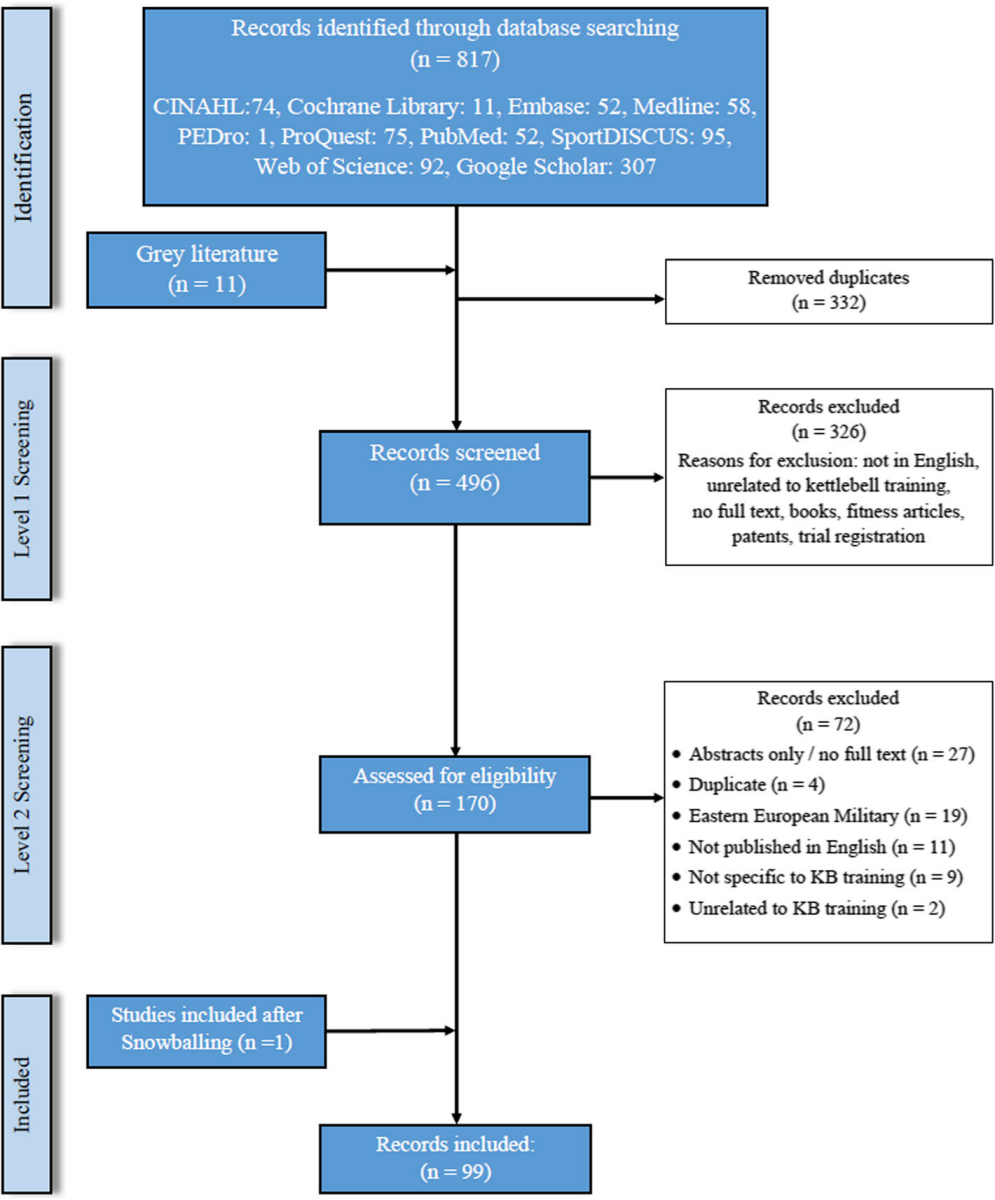
Study flow diagram (PRISMA-ScR flow chart)

**Fig. 2:**
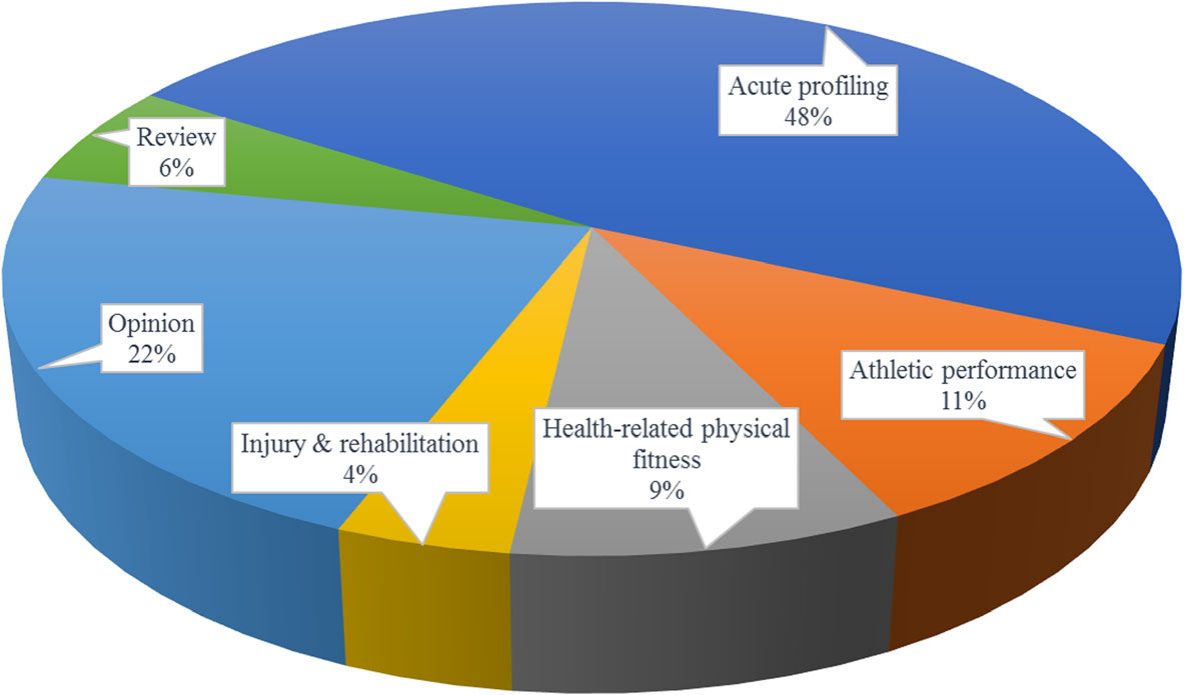
Kettlebell publications by category

The literature search yielded a total of 829 citations (Fig. 1). Three hundred and thirty-two records were removed as duplicates or not meeting the inclusion criteria. Upon completion of the title and abstract screening, 170 were potentially relevant and screened. Subsequently, 99 publications fulfilled the eligibility criteria and were included. Study flow diagram Fig. 1. Publications by category Fig. 2.

### Report characteristics (extent, range, nature)

**Fig. 3:**
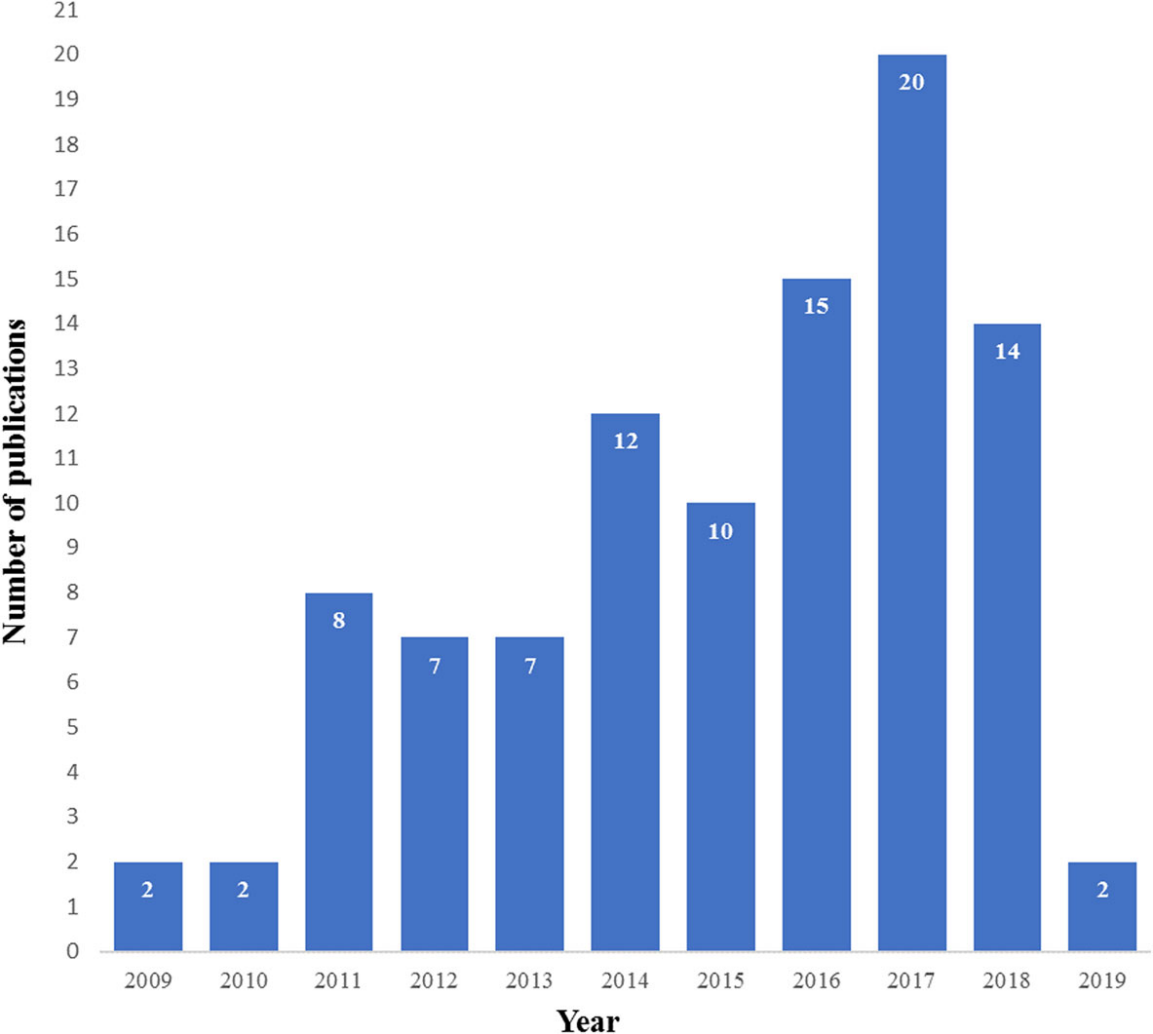
Number of academic publications by year involving kettlebells to February 1, 2019

**Fig. 4:**
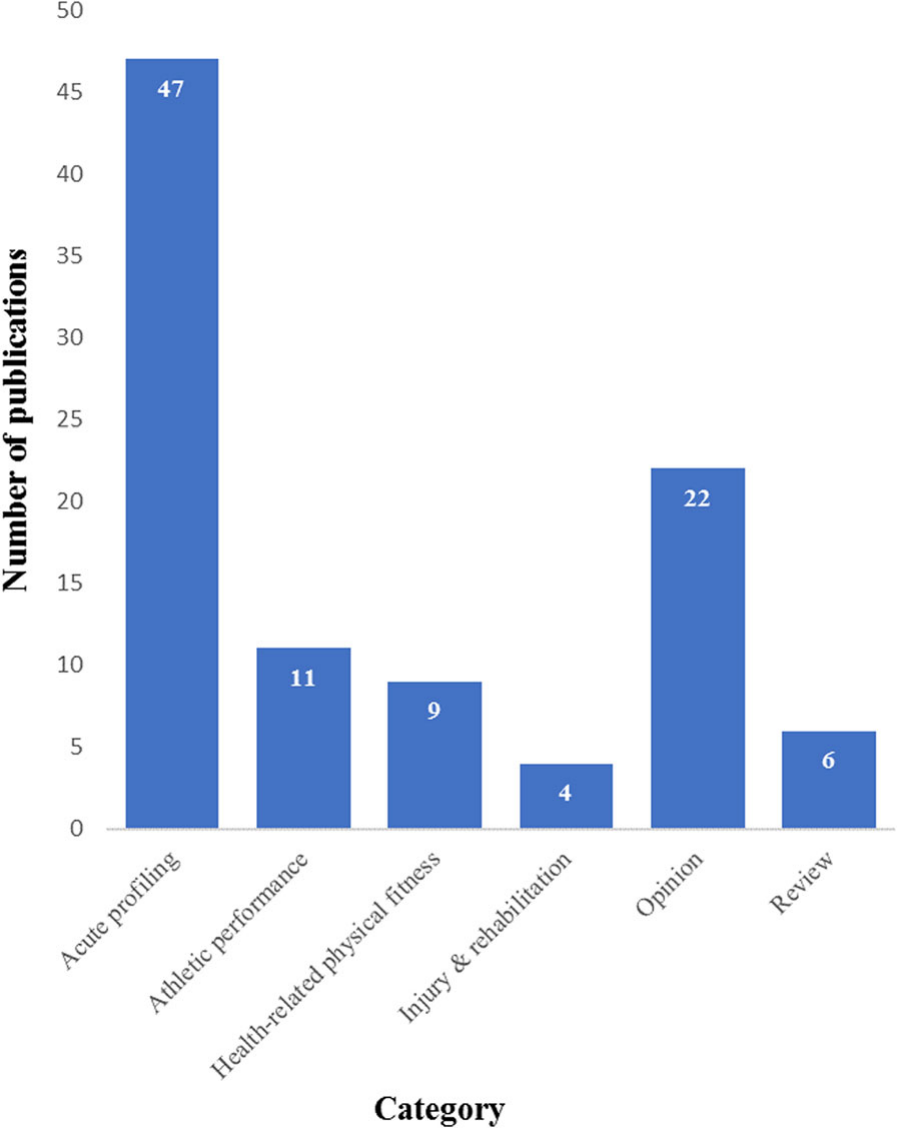
Kettlebell publications by category

**Fig. 5:**
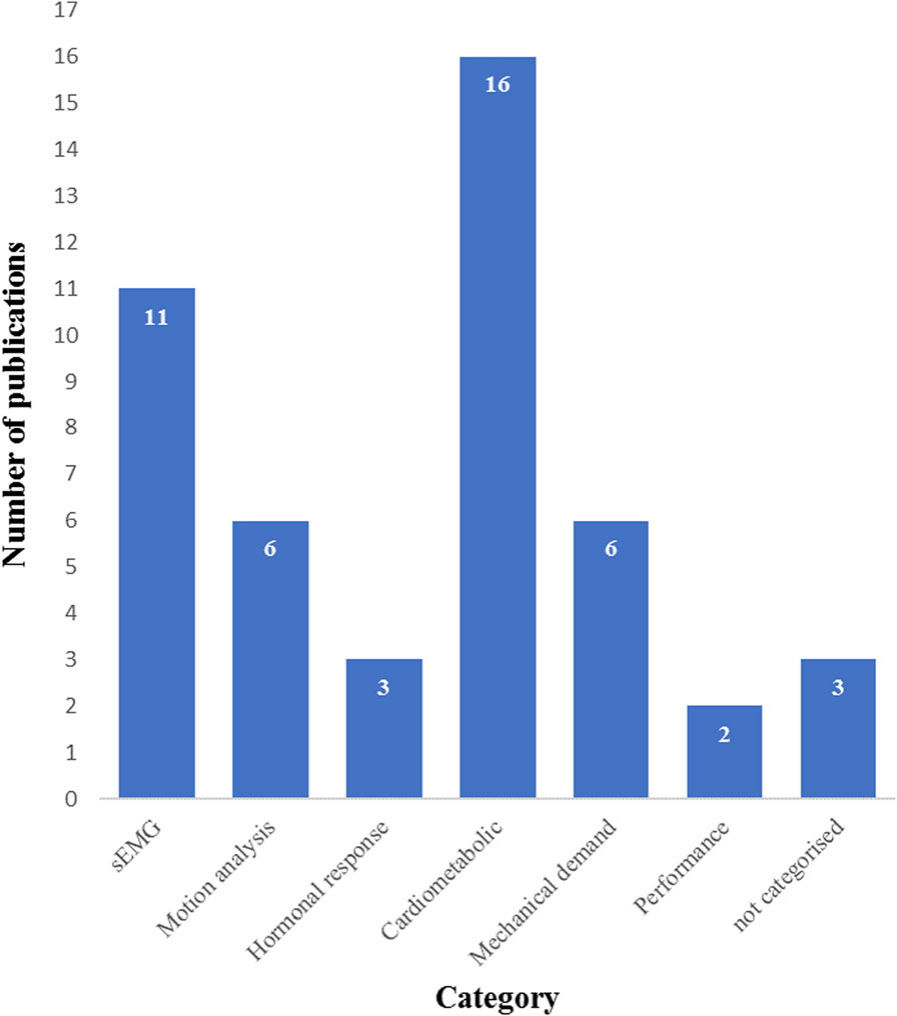
Acute profiling studies by category

The number of academic publications relating to the use of kettlebells has increased steadily since 2009 (Fig. 3) Sixty-eight (69%) of the publications were research studies, including 47 (70%) measures of acute training response and 21 (31%) longitudinal investigations. Two longitudinal trials involved clinical populations. Publications were categorised as ‘acute profiling’ [47], ‘Athletic performance’ [11], ‘Health related physical fitness’ [9], ‘Injury & rehabilitation’ [4], ‘Opinion’ [22] or ‘Review [6] (Fig. 4). Included in these were a Systematic Review, one Clinical Review, four Brief/Narrative Reviews, and 3 case reports from medical practitioners of injury attributed to kettlebell training. Acute profiling studies, which represent almost half of the publications, were further categorised based on outcomes: ‘sEMG’ [11], ‘motion analysis’ [6], ‘hormonal response’ [3], ‘cardiometabolic’ [16], ‘mechanical demand’ [6], ‘performance’ [2] or not categorised [3] (Fig. 5).

Fifty-four experiments and trials (79%) used healthy college-age participants, with participants in 62 studies (91%) recreationally active. In fifty-five studies (> 80%), participants were novices unfamiliar with kettlebell training, and almost half (*n* = 33) explicitly used hardstyle techniques and/or training principles described by Tsatsouline. Only 4 investigations (2 acute, 2 longitudinal) involved kettlebell sport. Of the 68 experiments and trials, 43 were published in peer-reviewed journals. The remainder were un-published conference presentations [5], Theses [9], Pilot studies [3], papers accepted for publication [4], and University publications [4]. Results described herein as significant where reported with *p*-values ≤0.05.

### Acute profiling

Forty-seven studies of acute response to kettlebell training were identified. Thirty-nine (83%) involved healthy college-age participants, 7 (15%) involved adults who were not of college-age, and 1 study did not report participant age. Twenty-one (47%) involved only males, 5 (11%) involved only females, and 19 (40%) had males and females. One study did not report gender. Only 1 study had participants who were not recreationally active. In 34 studies (72%), participants were not kettlebell-trained i.e. novices. Twenty-three studies (50%) explicitly used hardstyle techniques, and 3 (6%) investigated kettlebell sport. Training style/technique was unclear or not reported in 21 studies (43%). Three studies were given 2 category allocations [57–59] and 3 were uncategorised, deemed incompatible for synthesis [60–62].

### Acute profiling - surface electromyography (sEMG)

Eleven studies investigated sEMG. Muscles and regions investigated with exercise(s) and load(s) used are shown in Table 1. It appears that the TGU provides a roughly equal mechanical challenge to both shoulder girdles, one acting to stabilise the arm and kettlebell overhead and the other acting to support the body, through transitions from lying to kneeling and vice versa [66]. Among 14 common lower limb exercises used for therapeutic purposes, a two-handed swing was found to have the highest peak sEMG (115 ± 55%max), with greatest preferential excitation of the medial hamstring (Δ 22.5 ± 9.7% peak nEMG) [67]. A similar observation was noted with mean medial activity ≈10% greater than lateral activity across types of swing [63] with mean sEMG greatest during the hip hinge swing (35.74 ± 16.66), although the mean difference between styles was small (≈4–6%). In a dataset with large variation, excitation of the hamstring muscles was also observed to occur before the gluteal muscle in a one and two handed swing regardless of gender or range of movement [58].

**Table 1:** Study characteristics investigating acute sEMG with kettlebell exercise

Author	Participants						Observing (muscles / region)	Exercise	Hardstyle Sport ‘other’	Load (kg)	Comparator
	*n*	Age (yrs)	Weight (kg)	Gender	Active	Kettlebell proficient / novice					
Anderson et al. (2016)	16	25 ± 6	80 ± 8	M	Y	novice	trunk	2H swing	other	16	1H swing
Caravan et al. (2018)	33	22.2 ± 3.5	91.8 ± 8.0	M	Y	novice	serratus anterior	bottoms-up carry	other	12	deg. of abd^n^
Del Monte et al. (2017) [63]	14	30.1 ± 3.9	89.89 ± 19.72	M	Y	proficient	hamstring	2H swing	hardstyle	16–48	squat swing + dbl. knee bend
Dicus et al. (2018) [64]	21	21.4 ± 0.5 (F) 20.9 ± 0.7 (M)	69.7 ± 20.4 (F) 79.2 ± 12.1 (M)	M / F	Y	novice	deltoid + pec. Major	press	other	25% 1RM	dumbbell
Lim et al. (2018)	24	22.5 ± 3.3	70.82 ± 7.2	M / F	Y	novice	trunk + lower limb	2H swing	hardstyle	3	multiple
Lyons et al. (2017) [65]	14	21.5 ± 2.03	85.53 ± 8.11	M	Y	novice	trunk + upper + lower limbs	swing, clean + snatch	other	4.5–32	clean + snatch
Rajala et al. (2016)	9	21.4 ± 1.8	67.4 ± 9.6	F	Y	novice	lower limb	2H swing	other	4.5	OH swing
St-Onge et al. (2018) [66]	12	31.6 ± 8.2	72.4 ± 13.2	M / F	Y	proficient	shoulder girdle	Turkish get-up	hardstyle	8 (F) 16 (M)	none
Van Gelder et al. (2015) [58]	23	24 ± 1.97	61.8 ± 8.79 (F) 80.5 ± 10.0 (M)	M / F	Y	novice	gluteal + biceps femoris	2H swing	other	8–16	1H swing
Wu et al. (2019)	19	22.2 ± 1.1	76.0 ± 13.3	M	Y	novice	lower limb	squat + lunge	other	20	-
Zebis et al. (2013) [67]	16	23 ± 3	66.2 ± 7.4	F	Y	novice	hamstring	2H swing	hardstyle	12–16	multiple

*1H* one-handed, *2H* two-handed, *1RM* 1 repetition maximum, *OH* overhead, *abd*^n^ abduction, *dbl.* double

### Acute profiling – motion analysis

Seven acute studies investigated motion of joint segments or kettlebell trajectory (Table 2). Novices were found to perform a two-handed kettlebell swing differently to experts. Significant differences in joint segment angles and angular velocities at the hip and shoulder joint were reported during a two-handed hardstyle swing, with the order of movements reversed between conditions. During the up-swing (ascent), experts lead with the hips, then the shoulders followed. In the down-swing (descent), the arms drop first, then the hips flex. In novices, these joint segment sequences are reversed. Experts ‘hinge’ at the hips rather than squat (≈20^o^ greater hip flexion at the bottom of the swing and ≈15^o^ less knee flexion on the descent) stand up straighter (≈10^o^ more hip extension) and ‘swing’ the bell rather than ‘lift’ it (≈15^o^ less shoulder flexion at the bottom and ≈20^o^ less at the top) [68]. These findings are consistent with Tsatsouline [3] and with what is observed in practice.

**Table 2:** Study characteristics investigating acute motion analysis with kettlebell exercise

Author	Participants						Measurements	Exercise	Hardstyle / Sport / other	Load (kg)	Control / comparator
	*n*	Age (yrs)	Weight (kg)	Gender	Active	Kettlebell proficient / novice					
Back et al. (2016) [68]	7	32 ± 1 (exp) 30 ± 1.41 (beg)	70.5 ± 1.8 78.33 ± 3.86	unknown	Y	proficient	joint segment angle and velocity at the pelvis, hip, knee, ankle, shoulder, elbow and wrist	2H swing	hardstyle	16 kg	expert
Bullock et al. (2017) [57]	15	26.7 ± 4.3	77.6 ± 13.5	M / F	Y	proficient	cycle time + joint segment angle and velocity at the ankle, knee and hip	2H swing	other	12 - 20 kg	overhead + Indian club swing
Oikarinen et al. (2016)^a^ [69]	5	28–50	unknown	M	Y	novice	joint segment angles at the shoulder, elbow and wrist + segment angle and velocity at the lumbar	2H swing	other	16 - 24 kg	overhead
Ross et al. (2015) [70]	4	29–47	68.3–108.1	M	Y	proficient	horizontal and vertical displacement + velocity of the kettlebell	snatch	Sport	32 kg	none
Silva et al. (2017)^b^ [71]	1	25	72	M	Y	novice	joint segment ankles of arm-trunk, thigh-trunk and leg-thigh + time	OH swing	other	unknown	unstable surface
Van Gelder et al. (2015) [58]	23	24 ± 1.97	61.8 ± 8.79 (F) 80.5 ± 10.0 (M)	M / F	Y	novice	joint segment angle at the hip	2H swing	other	8 - 16 kg	1H swing
Zin et al. (2018)^b^ [72]	3	24 ± 0.82	70.2 ± 5.18	M	Y	novice	joint segment angles at the hip, knee and ankle	2H swing	hardstyle	4 - 8 kg	load

^a^thesis, ^b^conference paper, *2H* two-handed, *OH* overhead, *exp.* expert, *beg* beginner

Among a cohort of 23 novices, none of the participants obtained neutral hip position while performing any of the kettlebell swings, despite the notable availability of passive hip extension ROM, and cueing during the instructional sessions. Average terminal hip extension lacked a mean of 9.7° (± 7.8°) from neutral for both genders during the 2-handed swing. Of note, participants were only allowed to perform a maximum of 10 repetitions of each swing during the instructional session [58].

The kinematic similarities and differences between a swing to chest-height, a swing overhead, and an Indian club swing have been reported [57] although the clinical utility of these data is unclear. Cycle time for the overhead swing was 34% longer than the shoulder height swing and Indian club swing, with no differences in peak joint angles between the movements reported. No identifiable risk of injury from kinematic observation of the lumbar spine was identified when performing a two-handed swing to chest-height or overhead using a 16 and 24 kg bell, although reliability of these data is unclear [69].

Bell trajectory during a 32 kg single-arm snatch performed by four elite kettlebell sport lifters was reported to be similar between lifters and highly consistent within lifters. Anthropometric differences were suggested to most likely influence movement and performance efficiency [70]. On an unstable surface, reduction in trunk and knee flexion angles and reduced shoulder range of motion were reported during an overhead swing [71]; an expected compensation strategy to increase stability. Limited low-quality data suggested a possible trend toward decreasing mean flexion angles at the ankle, knee and hip with increasing bell weight among males novices using very light weights [72].

### Acute profiling – hormonal response

Very limited data is available regarding acute hormonal response to kettlebell training (Table 3). Changes in serum testosterone, growth hormone and cortisol have been observed following 12 rounds of two-handed swings with 16 kg [73]. Heavier bells had a larger effect on testosterone and cortisol when performing 12 min of swings in which workload was matched, however cadence was significantly different (8 kg at 42SPM Vs 16 kg at 21SPM) [75]. In practice, cadence typically remains consistent irrespective of kettlebell weight. A single 25-min kettlebell training session had similar effects on acute post-exercise glucose tolerance to high intensity interval running [74]. The clinical utility of these data is unclear.

**Table 3:** Study characteristics investigating acute hormonal response to kettlebell exercise

Author	Population						Measures	Exercise	Hardstyle / Sport / other	Format	Load (kg)
	*n*	Age (yrs)	Weight (kg)	Gender	Active	Kettlebell proficient / novice					
Budnar et al. (2014) [73]	10	19–30	78.7 ± 9.9	male	Y	novice	testosterone, growth hormone, and cortisol	Swing	hardstyle	30:30 × 12	16
Greenwald et al. (2016) [74]	6	24.3 ± 4.1	80.7 ± 10.2	male	N	novice	glucose tolerance	Circuit	other	25 mins	9–11.3
Raymond et al. (2018) [75]	10	19–43	82.2 ± 14.6	male	Y	novice	testosterone and cortisol	Swing	hardstyle	12 min 30:30	8–16

### Acute profiling – mechanical demand

Six acute studies investigated mechanical demands of kettlebell exercise (Table 4). Normalised to body mass, mechanical demands of a two-handed swing with 32 kg had the largest impulse (3.0 (0.2) N.s.kg^− 1^) when compared with peak back squat at 60% 1RM (2.1 (0.2) N.s.kg^− 1^) and jump squat at 40% 1RM (2.7 (0.4) N.s.kg^− 1^). A two-handed swing with 16 kg produced similar impulse to the jump squat at 0% or 60% of 1RM, and a 24 kg swing produced similar impulse to the 20%1RM jump squat [76]. The vertical jump has been used as a proxy for measuring power output, with the swing purported to be effective for improving activities associated with explosive hip extension, such as sprinting. A two-handed swing with 20% bodyweight produced a smaller average, peak and time-to-peak rate of force development, than a vertical jump [78], suggesting a lack of specificity to improve vertical jump performance.

**Table 4:** Study characteristics investigating mechanical demand of kettlebell exercise

Author	Participants						Measures	Exercise	Hardstyle / Sport / other	Load (kg)	Control / comparator
	*n*	Age (yrs)	Weight (kg)	Gender	Active	Kettlebell proficient / novice					
Lake et al. (2012a) [76]	16	24 ± 2	90.2 ± 14.4	M	Y	novice	impulse, peak and mean force and power to centre of mass, kettlebell displacement, peak and mean velocity	2H swing	hardstyle	16 - 32 kg	16, 24, 32 kg
Lake et al. (2014) [77]	22	28–41	75.2 ± 14.6	M	Y	proficient	impulse, mean force, displacement, magnitude, rate of work, phase durations and impulse ratio	2H swing	hardstyle	24 kg	snatch
Mache et al. (2016)^a^ [78]	25	22 ± 6 (F) 23 ± 2 (M)	66.4 ± 9.2 (F) 78.3 ± 8.5 (M)	M / F	Y	novice	peak, average and time to peak rate of force development	2H swing	other	≈20% BW	vertical jump
McGill et al. (2012) [79]	7	25.6 ± 3.4	82.8 ± 12.1	M	Y	proficient	peak and average muscle excitation, lumbar compression and shear force	1H swing	hardstyle	16 kg	swing with kime, snatch, bottom-up + racked carry
Mitchell et al. (2016)^a^ [80]	2	early 20’s	53 & 75	F	Y	proficient	position and orientation, joints and centres of mass of arm segments. Velocity and acceleration, forces and moments of the upper limb	OH swing	other	8 - 16 kg	8, 12, 16 kg
Ross et al. (2017) [5]	12	34.9 ± 6.6	87.7 ± 11.6	M	Y	proficient	ground reaction forces, velocity and temporal measures of resultant kettlebell force	snatch	Sport	24 kg	none

^a^conference paper, *1H* one-handed, *2H* two-handed, *OH* overhead, *BW* bodyweight

Vertical braking force with a 24 kg bell was reported to be approximately 25% greater during braking (down-swing) than acceleration (up-swing) during a two-handed hardstyle swing. Horizontally, the swing appeared to create approximately double the force and four-times the power of a single-arm hardstyle snatch using the same load. These data must be interpreted with caution however, as the start of the *propulsion* phase was an upright standing position holding the bell in front of the thighs and included the transition from upright standing to terminal backswing (bell between the legs). Despite the difference in vertical displacement of the bell (chest height vs overhead), force in the vertical direction was roughly equal to the swing, however the swing created approximately 40% more braking force. Approximately 15% more Work was performed in the down-swing during a hardstyle snatch than the swing. The swing has a significantly shorter braking phase (0.30s Vs 0.40s), larger Impulse ratio (time under tension: 21% Vs 14%) and propulsion (26% Vs 14%) than the snatch [77] likely attributable to the bilateral vs unilateral nature of the exercises.

During a single arm swing with 16 kg, a peak compression force of 3195 N at the lumbar spine was reported at the bottom of a swing, with an active bracing strategy described as the ‘kime’ increasing average compression by a further 1054 N [79]. A unique property of the kettlebell swing was reported as a *posterior* shear force (461 N), said to be so unusual that potential risks are unknown. The same study reported lumbar movement from 26^o^ of flexion to 6^o^ during a 2-handed swing with 16 kg.

During an overhead two-handed swing, a transition from tensile to compressive force at the shoulder was shown to occur approximately in the upper 30% of the bells’ arc in two females, with the majority of force and power reported to have been derived from the posterior chain musculature [80]. A peak resultant ground reaction force (GRF) of 1768 N (242) was reported among male amateur lifters, roughly equal to 2x mean bodyweight (87.7 kg ± 11.6 kg) [5].

### Acute profiling – performance

Two studies reported acute performance measures associated with kettlebell exercise (Table 5). One minute of two-handed swings with 16 kg was sufficient to induce fatigue (defined as a reduction in torque production) in the lumbar extensor muscles, but was significantly less than an isolated lumbar extension (MedX) exercise [81]. No significant interactions or main effects for any variable in countermovement jump performance were reported between kettlebell swings and kettlebell jump squats using a load equal to 20% bodyweight [82].

**Table 5:** Study characteristics investigating acute performance characteristics associated with kettlebell exercise

Author	Participants						Observing	Exercise	Hardstyle / Sport / other	Format	Load (kg)
	*n*	Age (yrs)	Weight (kg)	Gender	Active	Kettlebell proficient / novice					
Edinborough et al. (2016) [81]	10	20–25	79.94 ± 11.4	M	Y	novice	muscular fatigue (acute torque production)	2H swing	other	1 min continuous	16
Ros et al. (2016)^a^ [82]	7	19.14 ± 1.86	70.56 ± 7.25	F	Y	novice	post-activation potentiation on countermovement jump performance	2H swing	other	5 reps, 1 min rest, 5 reps, 3 mins rest	20% BW

^a^thesis, *2H* two-handed, *BW* bodyweight

### Acute profiling - cardiometabolic response

Establishing whether kettlebell training has the potential to increase aerobic capacity has been of interest to researchers. Sixteen studies reported acute cardiometabolic responses to kettlebell exercise (Table 6). The oxygen cost of completing as many two-handed swings as possible in 12 min (197 to 333 completed) with 16 kg was reported and compared with a graded treadmill test to exhaustion [85]. Classified as “hard” by ACSM standards, average HR (165 ± 13 b·min^− 1^ = 86.8 ± 6.0% HR_max_) was significantly higher than average VO_2_ (34.31 ± 5.67 ml·kg^− 1^·min^− 1^ = 65.3 ± 9.8% VO_2_max). At matched RPE, 10 min of two-handed swings compared with continuous treadmill running resulted in significantly lower VO_2_ (34.1 ± 4.7 Vs 46.7 ± 7.3 ml·kg^− 1^·min^− 1^), METS (9.7 ± 1.3) and energy expenditure (12.5 ± 2.5 Vs 17.1 ± 3.7 Kcal·min^− 1^). This was reported sufficient to increase aerobic capacity [88].

**Table 6:** Acute cardiometabolic response to kettlebell exercise

Author	Participants						Observing	Exercise	Hardstyle / Sport / other	Format	Load (kg)
	*n*	Age (yrs)	Weight (kg)	Gender	Active	Kettlebell proficient / novice					
Chan et al. (2018) [83, 84]	10	28.4 ± 4.6	95.1 ± 14.9	M	Y	proficient	VO_2_, HR, RER, V_E_, RPE	snatch	Sport	10 mins (cont.)	16
Duncan et al. (2015) [59]	16	23 ± 2.9	76.3 ± 14.7	M / F	Y	novice	HR, Bla	swing	hardstyle	2 mins, 40BPM & 80BPM	4–8
Farrar et al. (2010) [85]	10	20.8 ± 1.1	77.3 ± 7.7	M	Y	novice	VO_2_, HR	swing	hardstyle	12 mins	16
Ferreira et al. (2018) [86]	12	24.3 ± 7.0	74.1 ± 9.4	M / F	Y	novice	BP	swing	other	20 mins, 18 s/min	unknown
Fortner et al. (2014) [87]	14	18–25	66.5 ± 6.9 (F) 81.7 ± 3.0 (M)	M / F	Y	novice	VO_2_, HR, Bla, RPE	swing	other	4 mins, 20:10	4.5 (F) 8 (M)
Hulsey et al. (2012) [88]	13	19–27	73.0 ± 9.2	M / F	Y	novice	VO_2_, HR, METS, RER, ventilation, kcal, RPE	swing	other	10 mins, 35:25	8 (F) 16 (M)
Martin et al. (2012)^a^ [89]	8	28.5 ± 5.5	86 ± 15	M	Y	novice	HR, BP	swing	hardstyle	12 mins	16
Santillo et al. (2016)^a^ [90]	15	18–35	54.3–101.8	M / F	Y	novice	Bla	swing	hardstyle	volitional failure (RPE 15 or 95% HR_max_)	14–16 (F) 20–24 (M)
Schnettler et al. (2009)^a^ [91]	10	29–46	59.9–116.6	M / F	Y	proficient	VO_2_, HR, kcal, RPE	snatch	hardstyle	5 mins, 15:15	12–20
Schreiber et al. (2014)^a^ [92]	10	29–46	59.9–116.6	M / F	Y	proficient	HR, Temp, Bla, pain, thermal sensation, RPE	snatch	hardstyle	5 mins, 15:15	12–20
Šentija et al. (2017) [93]	11	25.9 ± 4.0	73.1 ± 21.1	M / F	Y	novice	VO_2_, HR, Bla, ventilation	swing	hardstyle	to exhaustion	Unknown
Thomas et al. (2014)^a^ [94, 95]	10	23 ± 4.4	75.45 ± 20.1	M/F	Y	novice	VO_2_, HR, BP, RER, METS, grip strength, RPE	swing + deadlift	hardstyle	3x 10mins, EMOM	8 (F) 16 (M)
Thomas et al. (2014) [94, 95]	10	25.3 ± 4.3	60.98 ± 16.1 (F) 93.5 ± 16.1 (M)	M / F	Y	novice	VO_2_, HR, BP, RER, kcal, RPE	swing + deadlift	hardstyle	3x 10mins, EMOM	8 (F) 16 (M)
Wesley et al. (2017) [1]	18	30 ± 9.6	68.2 ± 9	F	Y	proficient	HR, Bla, RPE	swing	hardstyle	15:15, ×10	8, 12, 16
Williams et al. (2015) [96]	8	21.5 ± 0.86	2.95 ± 11.62	M	Y	novice	VO_2_, HR, RER, TV, breathing frequency, V_E_, kcal	circuit	other	20:10, 4 mins, ×3	10–22
Wong et al. (2017)^b^ [97]	17	23 ± 1	74.1 ± 4.9	M / F	Y	novice	HR, BP	swing	hardstyle	30:30, ×12	8 (F) 16 (M)

^a^thesis, ^b^accepted for publication, *EMOM* every minute on the minute, *RPE* rate of perceived exertion, *BPM* beats per minute

Twelve rounds of two-handed swings produced significant mean increases in HR with each successive round (67 ± 0 at rest to 169 ± 5 bpm) and significant post-exercise hypotension at 10 (~ 4 mmHg SBP, ~ 3 mmHg DBP) and 30 (~ 4 mmHg SBP, ~ 3 mmHg DBP) minutes after exercise [97]. A reported resting mean HR of 67 ± 0 bpm in a group of 17 participants suggests challenges of reliability. Performing as many two-handed swings as possible in 12 min was also reported to be perceptually harder, with increasing feeling of heat stress, muscle pain and higher sustained HR compared to a kettlebell circuit workout completed at 90% 6RM [92].

A Tabata-inspired kettlebell circuit using a 2:1 work:rest ratio was compared to 1:8 (30s:4min) sprint interval cycling in “very active” males, with the kettlebell protocol proposed to be more attractive and sustainable [96]. The authors concluded that the high intensity kettlebell protocol would be effective in stimulating cardiorespiratory and metabolic responses, which could improve health and aerobic performance (mean VO_2_peak 29.1 ± 0.09 ml·kg^− 1^ min^− 1^ = 55.7% VO_2_max).

At a controlled work rate of 20 two-handed swings (40SPM) and 10 sumo deadlifts performed every minute on the minute, versus continuous cycling on an ergometer at 80 rpm, no significant differences were reported in any physiological (HR, VO_2_), subjective (RPE) or metabolic (RER, MET) response [94]. HR and RPE were significantly higher using the same 30-min kettlebell protocol when compared with treadmill walking at matched VO_2_, with no difference in RER, kcal.min^− 1^ and BP [95]. Both studies had male and female participants, with very large variation in anthropometrics.

No post-exercise hypotensive response was observed in normotensive individuals performing two-handed swings for 20 min [86]. A statistically significant attenuation in BP reactivity compared to control was reported, immediately following a cold pressor test, however the clinical utility of this phenomenon in practice is unclear. Significant reductions in post-exercise BP 120 min post-exercise were also reported following 12-min of discontinuous two-handed swings (88 to 486 swings completed), compared with a kettlebell circuit of 6 exercises among hypertensive or pre-hypertensive males [89]. Comparisons of effect are limited due to exposure bias, and a decrease of only 4 mmHg to reach clinical significance.

Jay’s VO_2_ snatch cadence test (cMVO2) [9] was modified by Chan [83] to simulate a kettlebell sport event and measure VO_2_ over 10 min. Increasing snatch cadence with 16 kg and multiple arm changes, was compared with a graded rowing ergometer with increasing power output. HR was comparable (≈175 ± 8-10b.min^− 1^) but mean peak oxygen consumption (37.5 ± 43.5 Vs 45.7 ± 6.6) respiratory exchange ratio (1.10 ± 0.060 Vs 1.18 ± 0.047) and minute ventilation (132.7 ± 19.2 Vs 157.1 ± 20.1) were significantly lower. VO_2_ response to the cMVO2 test was also reported to be significantly lower than the Bruce treadmill protocol (40.3 ± 2.2 Vs 49.7 ± 6.6 ml·kg·min^− 1^) among a small mixed gender group with very large variation in anthropometrics [91]. Mean VO_2_ was 31.6 ± 3.71 ml·kg·min^− 1^ however the range in HR (128-180 bpm), %VO_2_max [3, 69–83, 85, 86, 88, 89, 92, 94–97] and RPE [10–18] suggest these data may be poorly reliable.

An incremental kettlebell swing test (IKT) using increasing bell weight showed a strong correlation in peak oxygen uptake with the incremental treadmill test (3.27 ± 0.67 Vs 3.99 ± 0.71 LO_2_·min^− 1^, r = 0.92) [93]. Mean peak values for the IKT were significantly lower for VO_2_, HR, BLa and V_E_. It was reported that in most subjects, muscle fatigue rather than cardio-respiratory factors caused exhaustion in the IKT test. Clinical utility, validity and reliability of the IKT are currently unknown.

With respect to the modifiable factors of swing cadence, bell weight and rest periods, increases in kettlebell weight (8 kg,12 kg,16 kg) or cadence (32,40,48spm) were reported to significantly increase cardiometabolic demand (HR, RPE & BLa) [1]. It should be noted that kettlebell proficient participants reported that a cadence of 32spm was “unnaturally slow”, with the ballistic hip hinge eliminated and dynamic swinging motion becoming a static resistive motion. Researchers suggested that the resultant shoulder-dominant exercise likely inflated the physiological variables. In addition, swings have been reported to become perceptually harder with increasing bell weight [59], and reduced rest periods have significantly increased metabolic response when volume-matched with low load kettlebells [87]. The effect of different recovery strategies on lactate clearance following two-handed swings to volitional failure has been reported [90]. A statistically significant difference in clearance time and post-recovery performance occurred at ≈9-mins post-exercise, which is unlikely to be clinically meaningful.

### Acute profiling – ‘uncategorised’

Three publications (Table 7) were not categorised as the outcomes were unique and incompatible for synthesis. Small effect size reductions in pain pressure threshold (PPT) have been reported in lumbar and hip musculature following a Tabata-inspired (2:1) work:rest ratio, using a low-load, load-volume protocol [60]. A bilateral kettlebell carry was shown to be highly predictive of stretcher carry performance among Australian Army soldiers [61] with lean leg mass determined to be the most influential physical characteristic [62].

**Table 7:** Study characteristics investigating change in pain pressure threshold and task-related predictive test of a bilateral carry

Author	Participants						Observing	Exercise	Hardstyle / Sport / other	Format	Load (kg)
	*n*	Age (yrs)	Weight (kg)	Gender	Active	Kettlebell proficient / novice					
Keilman et al. (2017) [60]	60	25.12 ± 2.86	70.49 ± 13.32	M / F	unknown	novice	pain pressure threshold	2H swing	hardstyle	8 rounds, 20:10	8 (♀) 12 (♂)
Beck et al. (2016) [61]	73	43.4 ± 9.7 (F) 40.9 ± 10.2 (M)	67.2 ± 9.6 (F) 90.3 ± 12.4 (M)	M / F	Y	n/a	carry distance to volitional failure	farmer’s walk	n/a	at 4.5 and 5.0 km/hr	2 × 22
Beck et al. (2017) [62]	67	24–59	82.9 ± 15.7	M / F	Y	n/a	carry distance to volitional failure	farmer’s walk	n/a	at 4.5 km/hr	2 × 22

*2H* two-handed

### Long-term physiological response

Table 8 shows the outcomes from two randomised controlled trials using pragmatic hardstyle kettlebell training with older adults. Large effect sizes were reported in a mixed-gender group with Parkinson’s disease following 15 weeks of training [99]. Significant improvements were reported for the Timed Up and Go, Sit and Lift, elbow flexion and lower limb strength and torque measures compared to the Non-Periodic Activities Group which performed bodybuilding and stretching exercises. Very encouraging medium to large effect size increases in handgrip strength, back strength and sarcopenia index were reported in a good-quality RCT in women with sarcopenia [98]. Improvements in axial skeletal muscle mass and sarcopenia index were maintained at four weeks after cessation of training, with signification reductions in the same measures occurring in matched controls.

**Table 8:** Study characteristics investigating long term physiological response to kettlebell training

Author	Participants						Observing	Exercise	Hardstyle / Sport / other	Duration	Format	Freq /wk	Load (kg)	Control/ comparator	Randomised
	*n*	Age (yrs)	Weight (kg)	Gender	Active	Kettlebell proficient / novice									
Chen et al. (2018) [98]	33	66.7 ± 5.3	66.3 ± 12.1	F	N	novice	skeletal and appendicular muscle mass, body fat mass, sarcopenia index, pulmonary function, grip strength, isometric back strength, chronic proinflammatory cytokine concentration	× 11: swing, deadlift, goblet squat, squat lunge, row, single arm row, biceps curl, triceps extension, two-arm military press, Turkish get up	hardstyle	8	60 mins, 5 ex’s, 3 sets, 8–12 reps, 2–3 min rest	2	60–70% 1RM	Control	Y
Marcelino et al. (2018)	26	64.94 ± 9.29	74.85 ± 13.94	M / F (13 M, 4F)	N	novice	timed up and go, sit and lift, elbow flexion, 6-min walk, lower limb peak torque, Berg Balance Scale, static postural stability (COP displacement)	bottom-up, first stages of Turkish get-up, farmer walk, goblet squat, dead lift and swing	hardstyle	15	–	–	60–85% 1RM	body building and stretching exercises	N

### Long-term performance improvement

Table 9 shows the long-term performance improvements from kettlebell training. Changes in postural reaction time following kettlebell training have been reported in a good-quality RCT [105]. A basic low-volume, low-intensity program of kettlebell swings performed twice a week for 8 weeks, resulted in a large (109 ms) reduction in reaction time to perturbation. In a separate publication with the same participant demographics, relative reductions in mean musculoskeletal pain intensity of 57 and 46% in the low-back and neck/shoulder regions respectively were also reported [104].

**Table 9:** Study characteristics investigating mean long-term performance improvement from kettlebell training

Author	Participants						Observing	Exercise	Hardstyle / Sport / other	Duration (weeks)	Format	Freq/wk	Load (kg)	Effect	Effect size	Control/ comparator	Randomised
	*n*	Age (yrs)	Weight (kg)	Gender	Active	Kettlebell proficient / novice											
Ambrozy et al. (2017) [100]	40	27–32	unknown	F	Y	novice	hand speed, flexibility, explosive strength, strength endurance, agility, VO_2_, Wingate test	circuit (unknown)	other	8	60 mins	3	unknown	VO_2_ + 6.31 ml.kg^−1^.min^−1^ Average Power + 8.30 W Maximum Power + 20.53 W Bent arm hang + 13 s	Trivial (δ = 0.27, *p* ≤ 0.05) Trivial (δ = 0.10, *p* ≤ 0.05) Trivial (δ = 0.15, *p* ≤ 0.05) Moderate (δ = 1.36, *p* ≤ 0.05)	Minimally active	Y
Beltz et al. (2013) [101]	17	22.1 ± 2.80 (M) 21.5 ± 3.93 (F)	77.7 ± 10.86 (M) 64.5 ± 12.56 (F)	M / F	Y	novice	strength, aerobic capacity, body composition, flexibility, balance, and core strength.	circuit	hardstyle	8	45–60 min	2	unknown	Dynamic balance (PL) + 7.2 cm VO_2_ + 5.0 ml.kg^− 1^.min^− 1^ Leg press + 41.7 kg Grip strength + 1.7 kg Prone plank + 45 s	Moderate (SMD = 1.14, *p* ≤ 0.05) Trivial (δ = 0.33, *p* ≤ 0.05) Moderate (δ = 0.82, p ≤ 0.05) Small (δ = 0.42, *p* ≤ 0.05) Very large (δ = 1.32, *p* ≤ 0.05)	Y	N
Chen et al. (2018) [98]	33	66.7 ± 5.3	66.3 ± 12.1	F	N	novice	skeletal and appendicular muscle mass, body fat mass, sarcopenia index, pulmonary function, grip strength, isometric back strength, chronic proinflammatory cytokine concentration	×11: swing, deadlift, goblet squat, squat lunge, row, single arm row, biceps curl, triceps extension, two-arm military press, Turkish get up	hardstyle	8	60 mins, 5 ex’s, 3 sets, 8–12 reps, 2–3 min rest	2	60–70% 1RM	Sarcopenia index − 0.26 Handgrip strength (L) + 4.26 kg Handgrip strength (R) + 4.71 kg Back strength + 6.37 kg Peak expiratory flow + 0.79 L/s	Med/Lrg (δ = 0.79, *p* ≤ 0.05) Very large (δ = 1.2, *p* ≤ 0.05) Large (δ = 0.88, *p* ≤ 0.05) Large (δ = 0.83, *p* ≤ 0.05) Med/Lrg (δ = 0.79, *p* ≤ 0.05)	Y	Y
Elbadry et al. (2018) [102]	40	20.17 ± 0.4	75 ± 2.9	F	Y	novice	Standing LJ, softball throw, grip, LL strength, hammer throw	unknown	other	8	60 mins	3	unknown	Standing long jump + 11.37 Softball throw + 6.94 Handgrip strength (L) + 12.72 Handgrip strength (R) + 6.82 Static strength (BS) + 6.06 Static strength (LS) + 9.76 Performance (hammer) + 22.27	Medium (δ = 0.66, *p* ≤ 0.05) Huge (δ = 2.74, *p* ≤ 0.05) Huge (δ = 2.92, *p* ≤ 0.05) Large (δ = 0.94, *p* ≤ 0.05) Medium, (δ = 0.64, *p* ≤ 0.05) Very large, (δ =1.37, *p* ≤ 0.05) Very large (δ = 1.20, *p* ≤ 0.05)	Y	unknown
Falatic et al. (2015)	17	19.7 ± 1.0	64.2 ± 8.2	F	Y	novice	aerobic capacity (VO_2_)	Jay’s MVO2 snatch protocol	hardstyle	4	20 mins, 15:15	3	12	VO_2_max + 2.3 ml.kg^− 1^.min^− 1^	Medium (SMD = 0.72, *p* < 0.05)	circuit weight training	N
Holmstrup et al. (2016) [103]	18	18–25	unknown	F	Y	novice	Sprint performance	swing	hardstyle	8	10:10 × 8	2	9.1–13.7	Vertical jump + 2.4 cm Sprint -1 s	Small (SMD = 0.48, *p* > 0.05) Trivial (SMD = 0.04, *p* > 0.05)	N	N
Jay et al. (2011) [104]	40	44 ± 8	68 ± 11	M / F	N	novice	self-reported pain (neck/shoulders, back), strength (back, trunk, shoulder), VO_2_	swing	hardstyle	8	10–15 min, 30:60 to 30:30 × 10	3	8–16	Neck/shoulder pain VAS − 1.7 Low back pain VAS − 1.6 Back extension MVC + 19.6 Nm Trunk flexion MVC + 12.0 Nm Shoulder elevation MVC + 7.0 Nm VO_2_max + 2.9 ml.kg^− 1^.min^− 1^	Small (SMD = − 0.94, *p* ≤ 0.05) Small (SMD = − 0.89, *p* ≤ 0.05) Trivial = (SMD = 0.47, *p* ≤ 0.05) Trivial (SMD = 0.29, *p* > 0.05) Trivial (SMD = 0.39, *p* > 0.05) Trivial (SMD = 0.39, *p* > 0.05)	Y	Y
Jay et al. (2013) [105]	40	44 ± 8	68 ± 11	M / F	N	novice	postural reaction to external perturbation, vertical jump performance	swing	hardstyle	8	10–15 min, 30:60 to 30:30 × 10	3	8–16	Vertical jump + 1.5 cm Stopping time -109 ms	Medium (SMD = 1.5, *p* > 0.05) Large (SMD = − 2.87, *p* ≤ 0.05)	Y	Y
Lake et al. (2012)	21	18–27	72.58 ± 12.87	M	Y	novice	half squat 1RM and vertical jump height	swing	hardstyle	6	30:30 × 12	2	12–16	Half squat + 18 kg Vertical jump + 3 cm	Small (SMD = 0.82, *p*?) Small (SMD = 0.60, *p*?)		
Manocchia et al. (2013) [106]	37	40.8 ± 12.9	76.6 ± 14.4	M / F	Y	novice	transfers of strength and power + muscular endurance	> 20	other	10	60 mins	2	unknown	Barbell clean & jerk + 4.2 kg Barbell bench press + 14.2 kg	Moderate (SMD = 1.17, *p* < 0.05) Small (SMD = 0.63, *p* < 0.05)		
Maulit et al. (2017) [107]	31	23.1 ± 2.3	83.9 ± 13.8	M	Y	novice	strength and power	swing	other	4	4 sets × 5 reps to 6 × 4	2	10–12.5% MTP	Deadlift 1RM + 8.2 kg Vertical jump + 1.1 cm	Moderate (SMD = 1.17, *p* > 0.05) Small (SMD = 0.63, p > 0.05)		
Ooraniyan et al. (2018) [108]	30	18–23	unknown	M	Y	novice	lower limb strength, muscular strength	pistol squat, biceps curl, row, front raise	other	6	unknown	3	unknown	Lower limb strength Muscular strength ^a^measures not described^a^	Large (SMD = 0.40, *p* ≤ 0.05) Small (δ = 1.02, *p* ≤ 0.05) Moderate (SMD = 1.02, *p* ≤ 0.05) Large (δ = 0.99, *p* ≤ 0.05)		
Otto et al. (2012) [44]	30	19–26	78.99 ± 10.68	M	Y	novice	strength, power, and body composition	swing, accelerated swing, goblet squat	other	6	1–3 sets (3 × 6,4 × 4,4 × 6 then 4 × 6,6 × 4,4 × 6)	2	16	Vertical jump + 0.18 cm Back squat + 5.58 kg Power clean + 3.35 kg	Trivial (SMD = 0.05, *p* ≤ 0.05) Trivial (SMD = 0.18, *p* ≤ 0.05) Trivial (SMD = 0.18, *p* ≤ 0.05)		
Parasuraman et al. (2018) [109]	45	18–25	unknown	M	Y	novice	Max push-ups, plank endurance	various	other	6	45 mins	3	unknown	Max push-ups + 4 trunk endurance + 57 s	KB > CON, *p* ≤ 0.05		
Smith et al. (2014) [110]	28	20–29	unknown	M / F	Y	novice	post-activation potentiation and vertical jump	swing	other	8	×4–6 squat + × 5 CMJS	3	20–36	Vertical jump + 4.62 cm	Very small (δ = − 0.04, *p* > 0.05)		
Kramer et al. (2015) [111]	27	18–26	80.0 (M) 63.9 (F)	M / F	Y	novice	anaerobic power	various (×5)	other	4^a^	15:45 × 5 × 2	3	9–16	Mean Power Peak Power Rate of Fatigue	Trivial, *p* > 0.05		
Kruszewski et al. (2017) [112]	24	21.2 ± 1.2	89.83 ± 9.62	M	Y	novice	40yrd run, agility, CM jump, standing long jump, bench press	various	Sport	32	Complex	4	16–72	Training effects not reported			
Wade et al. (2016) [113]	17	30.4 ± 6.9	81.4 ± 12.4	M / F	Y	novice	USAF fitness, speed, power, and agility	swing	other	10	5 × 2 mins, 30s rest	3	6–14	Body fat (2.5%) Max push-ups [6] Max sit-ups [3] 1.5-mile run (12 s) Max grip strength (3.1 kg) Pro agility (0.1 s) Vertical jump (1.3 cm) 40-yard dash (0.4 s)	Small (δ = −0.37, *p* ≤ 0.05) Very small (δ = − 0.03, *p* > 0.05) Small (δ = − 0.47, *p* > 0.05) Medium/Large (d = − 0.79, *p* > 0.05) Small (δ = 0.10, *p* > 0.05) Small (δ = − 0.40, *p* > 0.05) Small (δ = 0.36, *p* > 0.05) Small (δ = − 0.40, *p* ≤ 0.05)		

^a^discontinuous

Moderate increases in upper limb endurance (bent arm hang time) have been reported in a moderate-quality trial [100]. Large improvement in trunk endurance (prone plank time), moderate improvements in dynamic single leg balance and leg press strength, and small improvement in grip strength, were also reported from a moderate-quality hardstyle kettlebell circuit performed twice a week for 8 weeks with young, heathy, active participants [101]. When compared to weightlifting training [44], there was no statistically significant difference between groups for the power clean, and only a small effect size difference in back squat strength was statistically significant. Changes in half squat strength and vertical jump height have been reported [114] although the effect size was small for a trained population. Small improvements in mean bench press 1RM and moderate mean improvement in barbell clean and jerk were reported from a comprehensive (> 20 exercises) pragmatic hardstyle kettlebell program performed twice a week for 10 weeks [106].

No conclusions can be drawn from a Pilot study comparing complex training protocols, in which kettlebell swings were compared with barbell back squats [110]. No statistically significant difference was found between groups on vertical jump performance, although reported to be “practically significant”. No significant difference was found in vertical jump and sprint performance in recreationally active females, although the training volume was described as inadequate [103]. Kettlebell swings using a ‘kettleclamp’ was reported to increase power and strength when compared with explosive deadlift training, however these conclusions were not supported by the data presented [107]. Limitations in study design prevent any conclusions being made from kettlebell training when compared with battle ropes [109, 111], on the physical performance of American Football players using kettlebell sport [112], male handball players [108], college females performing a hammer throw [102], or in military fitness training [113].

### Injury and rehabilitation

Based on a large differential in vastus lateralis (VL) to semitendinosus (ST) sEMG pre-activity during standardised side-cutting manoeuvres, a single case study was described of a female soccer player, retrospectively identified post-injury as a high risk of ACL rupture [115]. Risk was characterized by reduced sEMG pre-activity for the ST and elevated sEMG pre-activity for the VL, with a high-risk zone defined as one SD above the mean VL-ST difference [116] (Table 10). Ten months post-ACLR and standard post-surgical rehabilitation, the player was deemed ready to return to play despite persistence of the high-risk neuromuscular pattern. Based on the author’s previous work [67], a low-volume intervention (< 1000 swings, <25mins total time) performed over six weeks was reported to have reduced the player’s ACL risk profile from high to low.

**Table 10:** Single case study characteristics investigating kettlebell swings 10-months post-ACLR surgery

Author	Participants						Observing	Exercise	Hardstyle / Sport / other	Duration (weeks)	Format	Freq/wk	Load (kg)	Effect
	n	Age (yrs)	Weight (kg)	Gender	Active	Kettlebell proficient / novice								
Zebis (2017) [115]	1	21	unknown	F	Y	novice	differential in vastus lateralis to semitendinosus pre-activity (% of max EMG) Counter movement jump	swings	hardstyle	6	3–5 sets, ×20 reps, 20s rest (×10 sessions)	2	16–20	ST - 23% ↑ 61% BF - 26% ↓ 17% -0.3 cm

*ST* semitendinosus, *BF* biceps femoris

### Clinical opinion

Twenty-two opinion pieces from primary care clinicians and academics were identified, and 3 case reports of injury which a primary care physician had attributed to kettlebell training. The first publication was a summary of hardstyle training principles [117]. This was followed by a recommendation to include kettlebells in lower extremity sports rehabilitation [2] and a single case report incorporating kettlebells in the late stages of shoulder rehabilitation following rotator cuff surgery [118]. Two general articles about hardstyle training for rehabilitation purposes [119, 120], were followed by recommendations for using specific kettlebell techniques as a method of ‘functional training’, proposed to “mirror the challenges one faces in day to day activities” [121].

The TGU was described for patient self-management, to teach “the motor control needed for daily activities, occupation, and sports” [122] and specifically for integrating mobility, stability, symmetry (left, right, front, back), coordination, balance and strength [123], as a therapeutic exercise for injury prevention and performance enhancement [124], as a strength and conditioning tool for a variety of athletes [125], and as a component of kettlebell training to develop strength and power [126]. Only one article written for instructional purposes illustrates each of the ‘big 6’ techniques as descried by Tsatsouline [127]. Five kettlebell exercises have been individually described with proposed clinical or performance benefits; a modified swing [128], thruster [129], arm bar [130], reverse lunge with overhead press [131] and a lunge clean [132].

In sports, the use of kettlebells within program design has been described as a safe and effective modality that enhances the training experience [19] and was discussed in a point/counterpoint for inclusion in strength and conditioning [133]. A sample periodised program for the clean and jerk and snatch exercises within an athlete’s general conditioning for kettlebell sport has also been offered [134].

Three case reports of injury have been published. An onset of De Quervain’s tenosynovitis was attributed to repetitive trauma to the extensor pollicis brevis tendon [135], exercise induced non-traumatic rhabdomyolysis without complication [136], and a radial stress fracture [137]. Each case report appears to outline a training load error which may have accounted for the injury however, the potential influence of training load was not identified in any case. Broad risk management strategies appropriate for fitness professionals have also been described [138].

### Quality of evidence strength of recommendations

**Fig. 6:**
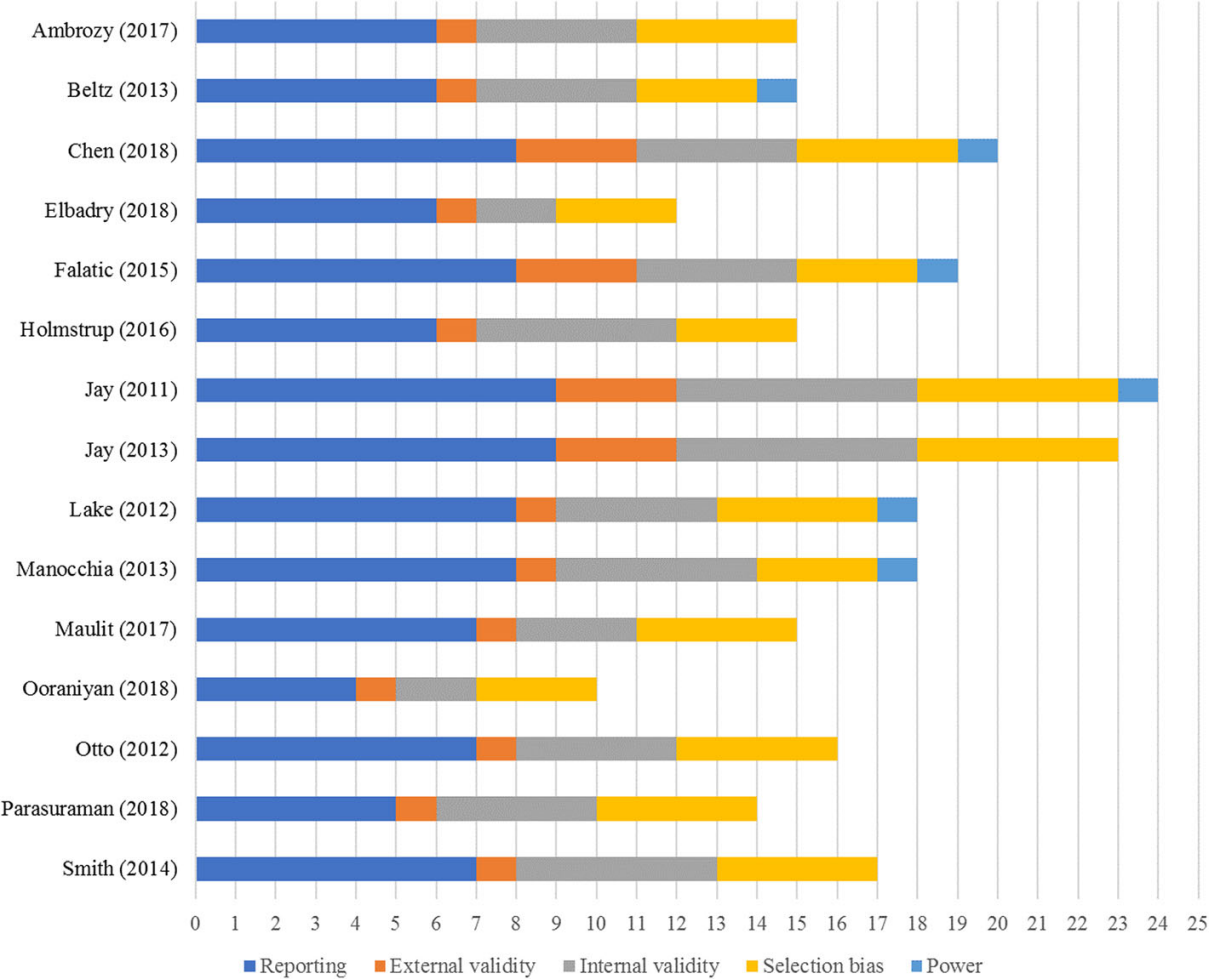
Modified Downs & Black quality assessment of Randomised Controlled Trials

Two reviewers independently evaluated randomised controlled trials using a modified Downs & Black quality assessment checklist [53]. Trials were excluded from quality assessment for the following reasons: i) single participant [115], ii) effects could not be attributed to only the kettlebell [113], iii) the trial was discontinuous [111] and iv) pre-intervention data was not captured [112]. The quality scores are illustrated in Fig. 6.

## Discussion

We conducted a scoping review which included 99 publications. The current body of evidence is represented by a small number of longitudinal studies, which are largely underpowered and generally of low methodological quality [56]. Three publications [104, 105, 108] from two studies had participants randomised to an intervention or inactive control. With high risk of bias, confidence in reported effects is low. Further research is very likely to have an important impact on our confidence in the estimate of effect. Trial descriptions of exercise interventions are suboptimal and no publication has used the Consensus on Exercise Reporting Template (CERT) [139] with only 1 RCT pre-registered. The validity of reported outcomes likely to have clinical utility, have yet to be established with repeated trials. Largely based on healthy college-age participants, the current body of research has limited applicability to clinical or high-performance athletic populations.

Our findings highlight a growing research interest in the effects of kettlebell training since 2009. There have been no adverse events reported during clinical trials, and no clear or quantifiable risk of harm from kettlebell training has been identified. It is unclear if the absence of reported adverse events is a true representation of kettlebell training or a limitation in reporting. Only 1 publication [127] illustrates how to perform each of the fundamental hardstyle exercises. Clinicians unfamiliar with kettlebell training wanting to prescribe them for therapeutic purposes, would be wise to consult with trained practitioners. Anecdotal reports of delayed onset muscle soreness, bruising and discomfort from repetitive impact force to the forearm among novices are not unusual. As a dynamic skilled activity using a free weight, it is advisable for a novice to receive appropriate instruction to mitigate avoidable error in execution or inappropriate loading.

Kettlebells are increasingly being used to perform exercises typically associated with other equipment, such as the one-arm bent over row and sumo squat. In these cases, the tool simply becomes a weight with a handle and the exercise (or potentially the outcome) not unique to the equipment. There may be instances where this is more desirable or necessary within a clinical context, however this becomes generalised exercise prescription and ‘training using a kettlebell’ rather than *kettlebell training*. Kettlebells are also being used to augment traditional exercises, such as hanging kettlebells by elastic bands to the end of an Olympic bar during a squat or bench press [37], which bear no resemblance to kettlebell training.

The differences between kettlebell sport and hardstyle could be summed up by a statement made by Valery Fedorenko in 2013, “It’s not about 5 or 10 sets of 10, its 1 set of 100; that’s the principle” [140]. In contrast, Jay described hardstyle training as “intermittent, high-powered work at maximal or supramaximal intensity in the correct ratio of work and rest” [9]. There are similarities and differences between kettlebell sport and hardstyle. For the primary care therapist or strength and conditioning specialist, there is no indication that one technique or style is better, more appropriate, or more effective than any other. Recommendation would most likely be based upon the provider’s experience with or exposure to kettlebells, and the person’s values, expectations and preference about an exercise program they may wish to engage in. Only 4 studies published in English have investigated kettlebell sport. Two involved acute biomechanical analysis of kettlebell exercises [5, 70], 1 involved the development of a kettlebell snatch protocol for kettlebell sport that could be used in the laboratory [83], and 1 was a University study showing medium to huge effect size changes in standing long jump, strength and throw performance, although with high risk of bias the results are unreliable [102].

The U.S. Department of Energy “Man Maker” protocol was described by Tsatsouline as “*alternate sets of kettlebell swings to a comfortable stop, with a few hundred yards of easy jogging for active recovery”.* Performed twice a week for an arbitrary time of 12 min, it was recommended that people also complete 2 days each week of 5 min continuous TGUs. The program would continue until they could perform 100 single-arm swings < 5 mins and 10 TGUs < 10 mins at a target weight. In the research literature, the Man Maker challenge was first cited by Farrar [85] as a “popularly recommended kettlebell workout”, however the study protocol used was 12 min of *continuous* two-handed swings. The same 12-min continuous format was subsequently used to measure blood pressure response [89] and later compared to a high-resistance circuit workout [92]. Whilst hardstyle techniques were cited, these studies illustrate an evolution in the literature away from the principles and practices described by Tsatsouline, based upon researcher’s interpretation of training practices.

Due to the variety of ways in which an exercise prescription could possibly include kettlebell exercises for clinical and athletic populations, it is vital that the exercise professional has a clear idea of the acute stresses imposed on the body by this form of exercise prior to its utilisation. An initial understanding of these acute stresses is being provided by studies assessing the acute hormonal, kinetic, kinematic, cardiometabolic and electromyographic responses to kettle bell exercise in a range of populations.

Surface electromyography (sEMG) is a popular research tool which records the electrical potential of skeletal muscle, with a wide variety of clinical and biomedical uses. Within rehabilitation sciences, EMG signals are collected as participants perform the activity under investigation, frequently using different loading conditions. Common methodology involves the comparison of EMG amplitudes, with researchers making conclusions based on the neuro- and electrophysiological correlation with muscle force. Hypotheses may be made regarding potential longitudinal adaptations in the characteristics and performance of skeletal muscle, such as strength and hypertrophy. However, conclusions cannot be made about muscle activation, force and mechanisms of force production, or inferences made from longitudinal outcomes based solely on sEMG amplitude [141] The use of unconventional exercises [64, 65] adds further complexity to the interpretation. With execution of a swing influenced by so many variables, it is likely that the differences between swing types may not be clinically meaningful, although considered important within their own discipline.

The difference in movement pattern between expert and novice performing a two-handed hardstyle swing [68] is consistent with 1^o^ of hip extension observed at the top of a swing in kettlebell-trained subjects [79] and with what trainers report in practice. The skill acquisition of a hardstyle swing appears clear and consistent, however its utility in clinical practice is unclear. The observed difference between expert and novice is likely to apply to other kettlebell exercises, thus the experience of participants in research studies should be considered when assessing validity of findings, and the generalisability of outcome data to other populations. Other factors likely to influence outcomes include kettlebell specific differences such as training style, bell weight and swing cadence, and factors common to other training modalities such as work-to-rest ratio, peripheral and central fatigue. Each of these should be assessed when prescribing kettlebell exercises and their relative importance established for clinical populations on a case-by-case basis.

There is no indication that one type or style of swing has greater clinical utility than another. No data suggests that someone performing a swing counter to the prescriptive hardstyle pattern, is at increased risk of harm. When performing a hardstyle swing in practice, much emphasis is placed on the production of power (in the horizontal plane) and of developing “power-endurance” [3] however, no published data currently exists which quantifies or validates these claims. The potential for using movement(s) associated with kettlebell training for therapeutic purposes has not been investigated.

Although limited, ground reaction force data is clinically helpful, particularly where the mechanical demands of a kettlebell swing are compared to other commonly used exercises, or where some objective quantifiable loading of tissues is indicated. Large increases in ground reaction force relative to bodyweight [5] may be of interest to clinicians where manipulation of lower limb load is needed, such as with symptomatic knee osteoarthritis. The load influence from kettlebell training on specific joints, or with musculoskeletal conditions more generally, remains unknown and warrants investigation.

Lumbar motion, compression and shear force data during a kettlebell swing offer meaningful information, albeit limited to a single study [79]. These data are encouraging, that in the absence of spinal pathology, mechanical loads through the lumbar spine during a 16-kg two-handed hardstyle kettlebell swing are low and not indicative of increased risk of harm. Indeed, compression loads were reported below the National Institute for Occupational Safety and Health action limit, and half that of lifting 27 kg on an Olympic bar. Resultant spine loads were described as “quite conservative” and “not be problematic”. How these forces might change with increasing kettlebell weight is not known and clinicians should be cautious not to assume they remain low. Biomechanical modelling identified a unique posterior shear force in the lumbar spine during a kettlebell swing. Whether this is a consistent feature across individuals remains to be seen, and the potential effect on pathological presentations such as spondylolisthesis, a pars interarticularis defect, or osteoporosis is unknown. More common resistance training exercises such as a barbell deadlift produce an anterior shear force at the level of L4/5, with forces of much larger magnitude reported among competitive power lifters [142]. Until further data is available, clinicians would be wise to use caution if considering a kettlebell swing with someone who has a significant or unstable lumbar spine pathology. Additionally, among kettlebell-trained subjects, the lumbar spine was reported to flex approximately half full range (up to 26^o^) at the bottom of the swing [79].

Greater time-under-tension (impulse) may support the premise of enhancing power endurance, however the clinical utility of impulse when compared with other forms of resistance exercise is unclear [76]. The manipulation of resistance training variables is widely considered an essential strategy to maximise muscular adaptations, and guidelines exist in relation to volume load to maximise muscle hypertrophy. No consensus however currently exists for a metric of volume load in resistance training [143] and kettlebell weight is likely to be well below an intensity threshold sufficient to stimulate anabolism. Any difference in impulse per repetition compared with back squat and jump squat [76] are unlikely to be clinically meaningful when compared with the kettlebell weight and number of repetitions performed in a training session. Further research is required to better understand the mechanical demands of kettlebell training, which may involve several hundreds of repetitions and multiple exercises.

The clinical utility of reduced torque in the lumbar extensor muscles following swings is unclear [81]. Consistent with temporal and kinetic data [78], no significant difference in countermovement jump performance [82] suggests that kettlebell swings are unlikely to provide any meaningful benefit to jump performance. Change in pain pressure threshold may be used in clinical practice, however there is no suggestion that this phenomenon would be unique to a kettlebell swing, or that change in pain pressure threshold following kettlebell swings [60] has a clinically meaningful effect. Loaded carries are also not unique to kettlebells, so the utility of carry data for clinical practice in relation to the specific prescription of kettlebell exercises remains limited [61, 62]. Kettlebell carries however (rack, bottoms-up, overhead, suitcase) have been proposed as good exercise to increase trunk stiffness and reduce “energy leakage” when transmitting power generated by the hips, to sporting and daily living tasks involving pushing, pulling, lifting, carrying, and torsional exertions [144]. These principles do have clinical utility but have not been investigated.

Kettlebell training appears to induce a cardiometabolic response sufficient to improve cardiovascular fitness [1, 59, 84, 85, 87, 88, 91–96] provided that the dose (kettlebell weight, volume load and work:rest ratio) is appropriate for the individual and sufficient to provide a supraphysiological load. Effects have often been over-reported, and reliable clinically meaningful effects remain to be demonstrated in a high quality randomised controlled trial. Many of the same investigations have also demonstrated kettlebell training produces a lower peak VO_2_ when other physiological and metabolic variables are matched [83, 88, 93, 95]. These data are consistent with suggestion that hardstyle kettlebell training is not the most effective form of exercise for improving cardiovascular capacity. Physiological mechanisms for the pressor response (disproportionately elevated HR when compared to oxygen consumption during resistance training) have been proposed, however these claims have not been validated in practice [8].

A basic kettlebell swing protocol has shown to produce a similar cardiometabolic demand to other forms of physical activity such as walking [95] and cycling [94]. For someone who is home-bound with a cardiometabolic condition requiring a significant exercise stimulus, a single kettlebell exercise may be a suitable alternative to walking and cycling. The long-term cardiometabolic effects of kettlebell training remain equivocal. Further investigation with high quality trials will help clinicians better understand the potential for kettlebell training to improve cardiorespiratory fitness in clinical populations.

Expert hardstyle practitioners performing a swing to chest-height, typically have a cadence of 40 swings per minute [1]. Swing cadence for the American swing and a ‘low swing’ in Sport training would be lower, and cadence within Sport is typically well-controlled by the individual. Further research using a kettlebell swing should ensure that cadence reflects the practice or discipline it seeks to inform or make explicit why deviations from normal practice are being investigated.

Encouraging for the primary care clinician are improvements in axial skeletal muscle mass, sarcopenia index, grip strength and back strength, from a good-quality randomised controlled trial with sarcopenic elderly females [98]. During the 8-week training period controls had significant reductions in muscle mass and grip strength, with significant increase in visceral fat area. These data need to be reliably repeated with trial descriptions using the Consensus on Exercise Reporting Template to facilitate replication and to inform clinical practice. With an ageing population and increasing importance placed on identifying effective strategies to maintain musculoskeletal fitness, independence, self-confidence and quality of life in primary care, kettlebells could be an ideal prescription for older adults. Resistance training is considered the best countermeasure for preventing sarcopenia, there are no non-responders in the older population [145–147], and kettlebells have been recommended for their ease of teaching, cost effectiveness and being less intimidating to use that other resistance equipment.

In research and clinical practice, hand grip strength is one component of the algorithm used to make a clinical diagnosis of sarcopenia [148] and improvements in grip strength from kettlebell training have been reported [98, 101, 102, 113]. This is encouraging as poor hand grip strength is a consistent predictor of falls and fractures in both sexes among older adults [149], and an independent predictor of all-cause mortality and cardiovascular diseases in community-dwelling populations [150–153]. Lower limb muscle strength is also independently associated with elevated risk of all-cause mortality, regardless of muscle mass, metabolic syndrome, sedentary time, or leisure time physical activity [154]. Significant increases in lower limb strength [101, 108], dynamic single leg balance [101], and reductions in postural reaction time [105] from kettlebell training, represent an interesting constellation of effects. If each of these are achievable for older adults, kettlebell training may have the potential to reduce falls risk, improve physical function and increase independence. Further research in this are appears warranted.

Similar data from pragmatic training among elderly adults with Parkinson’s disease is equally encouraging [99], especially following a recent systematic review of resistance training for Parkinson’s Disease which reported that it is hard to establish a correlation with improved physical parameters and quality of life [155]. Qualitative data has not been reported and so the potential uptake more broadly of kettlebell training with these older populations in clinical practice remains unknown.

The potential for kettlebell training to improve measures of physical performance has received the most research interest to date. There is little evidence however to suggest that kettlebell training specifically, is likely to provide athletes with any marked improvement in sports performance, with claims to the contrary remaining conjecture. Limited data comparing effects of kettlebell training with weightlifting [44] showed only a small statistically significant effect size difference in back squat strength, however these data are unreliable due to a large exposure bias in favour of the weightlifting group (80% 1RM vs 16 kg kettlebell and training 2/3 of the measures). That weightlifting training with an exposure bias did not significantly outperform the kettlebell training, as might have been expected, perhaps warrants further investigation.

Changes in half squat strength and vertical jump height were reported in another study [114] although the effect size was small for a trained population. In a third comprehensive kettlebell training program [106], confidence that the reported improvements in bench press 1RM and barbell clean and jerk are representative of the true training effect is low, due to a very large variation in participant age, training history and baseline physical capacity. The addition of a reverse lunge with single arm snatch in the fourth microcycle (80–85% RPE) and TGU in the fifth microcycle (85–95%), are technically complex exercises. Questions of external validity may have been addressed had a CERT being reported. Combined, these data do provide limited support for using kettlebells to improve health-related physical fitness. High-quality randomised controlled trials are needed to increase confidence in the true effects.

Numerous musculoskeletal conditions influence the functional capacity of the upper limb and shoulder girdle. The clinical impact of improving bent-arm hang time from kettlebell training [100] is unclear, and these data should be used with caution due to risk of study bias. Similarly, improvements in trunk endurance, dynamic single leg balance, leg press strength and grip strength among young healthy individuals should also be repeated to establish validity of these effects [101]. A notable inclusion of this study was the reliability assessment of Jay’s cMVO2 test [9] reported to be R = .94. For kettlebell practitioners this may be very helpful, however this test likely has little value in clinical practice. A significant practical limitation of the test is the need for the participant to have a high degree of proficiency in executing the snatch, making it only suitable for well-trained kettlebell practitioners.

Large relative reductions in self-reported musculoskeletal pain intensity following kettlebell training [104] have been widely cited, although the effect size was only small, and within-group change did not reach a minimum clinically importance difference of 2 points on a numeric pain rating scale. In addition, participants did not need to have pain to enter the study. Claims of reducing musculoskeletal pain in clinical practice are not currently well supported.

Kettlebell swings have been proposed to reduce the risk profile of ACL injury [115] due in part to the high excitation of the medial hamstrings [67]. Kettlebell swings may have a place in a person’s training and rehabilitation, however there is insufficient evidence at this time to warrant their inclusion in clinical practice guidelines. Further research pre- and post-ACL injury is required before clinicians should recommend kettlebell swings as a primary means for managing risk of injury and return to sport. Unique to hardstyle kettlebell training, the TGU is practiced widely and recommended with numerous claimed benefits, with clinical case studies now emerging [156]. As a loaded floor transfer exercise which is scalable, the TGU has a range of potential uses in clinical practice from geriatrics to athletes, but to date has been almost entirely overlooked by research investigation. A recent descriptive analysis of shoulder muscle excitation [66] provides some insight into its potential use in a rehabilitation context, specifically for the upper limb and shoulder girdle, but its use remains anecdotal and unsupported in the absence of clinical trials.

In each reported case of kettlebell injury during training [135–137], a loading error may have been the primary cause, so clinicians should have little cause for concern in using them. For example, in the case of a female kettlebell sport competitor with a radial stress fracture, it is stated that “she had recently increased her frequency and intensity of kettlebell workouts” with the onset of symptoms commencing after performing a single arm snatch with a 24 kg bell. The potential for kettlebells to improve strength and cardiorespiratory fitness, or reduce musculoskeletal pain is not well supported by the existing body of evidence. Kettlebells could be used clinically to address pathological pain conditions using inhibitory learning mechanisms and expectancy violation, however that cannot be unique to the kettlebell. If the clinical goal is to maximise exercise-induced hypoalgesia, current evidence does not indicate that kettlebells would be most effective [157].

Significant small-to-moderate effects have been observed in a range of physiological parameters in active, healthy, college-age populations, which may represent opportunities for the prescription of therapeutic exercise prescription within primary care. Applying the GRADE [56] criteria to the current body of evidence however, confidence in reported effects remains low, with strength of recommendation weakly in support of improving physical function or performance. This is likely due to participants being largely under-dosed in experimental conditions. Within primary care, the potential benefits of kettlebell training remain untested.

Clinical guidelines [158] are not based upon studies in populations undergoing rehabilitation, and prescription for any form of musculoskeletal rehabilitation are currently absent. One third of the 15 citations are fitness publications, and 2 are clinical opinions from authors who may not have received any formal kettlebell training. Contraindications/precautions refer only to ‘resistance exercise in individuals with and without cardiovascular disease’. Physical examination recommendations are unrelated to prerequisite physical capacity or movement competency which may be required in order to execute a kettlebell exercise, and treatment summary recommendations from the fitness industry may be inappropriate for individuals experiencing pain, of have functional limitations from disease or disability.

In addition to the clinical review [158], 5 further reviews of varying breath and utility have been published to date. The first review in 2014 discusses the effects of kettlebell training on measures of strength and power, cardiovascular measures, and biomechanics [159]. This was followed by a systematic review in 2015 of the effect of kettlebell training on strength, power, and endurance, which included 5 studies [160]. A brief review in 2016 had a broader scope, which included 14 publications to summarise the efficacy of kettlebell training for increasing muscular power, strength, muscular endurance, and aerobic capacity [161]. A 2017 mini narrative review sought to review the implications of kettlebell training for exercise programming [162] and finally, a 2018 review compared kettlebell training as a method of resistance training on hypertrophy, strength and power, to a range of other resistance training methods [163].

There has been growing interest in, and academic exploration of, the effects of kettlebell training in the last 10 years, however the current body of evidence is challenged by limited internal and external validity, high risk of bias due to lack of blinding, and underpowered small sample sizes. Additionally, less than optimal study design, flaws in reporting, and inferences from a typically homogenous population of your healthy participants unfamiliar with kettlebell training, have limited application to conditions commonly managed in primary care. The existing body of evidence provides little guidance to inform the prescription of kettlebell exercises in clinical practice. Our review highlights only that insufficient data currently exists to strongly support claims of improvements in performance, or measures of health-related physical fitness from kettlebell training, rather than there being evidence of no effect.

### Directions for future kettlebell research

For the clinician and therapist in primary care, there are many gaps in the research literature for integrating kettlebells into practice. A common language is needed and clear standards for clinicians and researchers to follow in teaching, performing and dosing exercises, and in measuring and reporting effects. Updated clinical practice guidelines are needed which better reflect the populations and health conditions managed in primary care. Below are our suggestions to future researchers in areas which may have clinical utility.

#### Pathological pain

Future research could investigate the utility of using kettlebells to help people who have pain, arguably the most common presentation in primary musculoskeletal care. In combination with other approaches, movement and loading (mechanotherapy) is often used to modulate non-nociceptive pathological pain states. As a tool which can replicate ADLs such as lifting and carrying tasks, the versatility of a kettlebell makes it a useful tool within a clinic setting and could be a more effective option within an active rehabilitation plan than current options. Other common musculoskeletal conditions for which kettlebell training may be suitable include shoulder instability, rotator cuff related shoulder pain, gluteal and elbow tendinopathy, and non-specific low back pain.

#### Post-surgical rehabilitation

The hallmark of post-surgical rehabilitation in clinical care is the progressive loading of tissues and restoration of movement and function. Future research could investigate the utility of using kettlebells for a wide range of post-surgical conditions compared with existing protocols and conventional equipment.

#### Knee osteoarthritis

Ground reaction force during a kettlebell swing suggests that this exercise could be an effective means of improving function and reducing the pain associated with knee osteoarthritis. An activity which commonly aggravates arthritic knees is ascending and descending stairs, however vertical ground reaction force only reaches 1.4–1.6x bodyweight on the descent [164]. It appears that a kettlebell swing has the potential to far exceed normal ground force when using stairs and could provide sufficient stimulus for a positive adaptation. Future research could examine the utility of a kettlebell swing program to positively influence symptoms and delay the need for surgery. With the same clinical rationale, future research could investigate the utility of a similar protocol to restore function following knee arthroplasty.

#### Mechanical demands and training load

Clinicians need to better understand the potential influence that variations in gender, age and training history may have on the mechanical demands of kettle bell training, and how these factors may influence the therapeutic prescription of kettlebells and training loads. More research is required beyond convenience samples of healthy college students, with clinical practice guidelines providing data relating to appropriate internal and external training loads, in different populations and health conditions. Claims of hardstyle training relating to the development of ‘power endurance’ and the horizontal vs vertical components need to be tested, and the validity and reliability of those measures established.

#### Pragmatic kettlebell training

More research is required which uses a pragmatic approach to training with kettlebells. Whilst single exercises such as the swing may have clinical and research utility, a pragmatic approach which is more inclusive of other exercises would be helpful. Primary care clinicians would benefit from a better understanding of kettlebell training in the context of clinical practice, rather than the use of isolated exercises. As primary care practitioners are encouraged to promote physical activity generally and resistance training specifically, it is incumbent to understand its effectiveness at a population level compared with other community-based exercise options.

#### Health-related physical fitness

Finally, promoters of hardstyle kettlebell training suggest that it can improve measures of health-related physical fitness. Future research is required to validate these claims and to establish associated training stimuli and effect sizes.

### Limitations

There are some limitations to our scoping review methods. Firstly, scoping reviews have inherent limitations because the focus is to identify knowledge gaps, inform future research, and identify implications for decision-making [50]. Formal reporting of methodological quality was limited to only randomised controlled trials. The eligibility criteria defined by the *context* (evidence-based practice: research evidence and clinical expertise) precluded commentary from non-clinical, non-academic sources. Potentially valuable sources of information exist within the fitness industry and subject matter experts e.g. certified kettlebell trainers, with this source of information typically disregarded when synthesising higher levels of ‘evidence’ to inform clinical practice. A priori protocol was not developed. The review was limited to documents written in English to increase its feasibility. The data was abstracted and processed by a single reviewer. Whilst the literature was comprehensive, it is possible that some publications may have been missed. Since this is a rapidly evolving and emerging field, we expect that new publications fulfilling our inclusion criteria will be released in increasing numbers, highlighting a potential need to update our review and/or to conduct systematic reviews on more specific kettlebell related questions in the near future.

## Conclusions

Significant small-to-moderate effects from kettlebell training have been observed in a range of physiological parameters among healthy, physically active college-age cohorts. Significant clinically meaningful moderate to large effects have been reported from pragmatic hardstyle kettlebell training in older adults with Parkinson’s disease and older females with sarcopenia. While confidence in reported effects however remains low to very low, and strength of recommendation only weakly in support of kettlebell training until effects have been reliability repeated in high-quality trials, the opportunities within primary care remain promising.

The current body of evidence is challenged by limited internal and external validity, high risk of bias primarily due to the lack of blinding, underpowered small sample sizes and participants largely under-dosed in experimental conditions. Less than optimal study design, flaws in reporting, and inferences from a typically homogenous population, have limited applicability to pathological conditions in primary care, or more broadly to clinical populations.

Within primary care, the potential benefits of kettlebell training are currently based on conjecture, with further research and high-quality clinical trials needed to make a shift from practice-based evidence to evidence-based practice. Presently, the therapeutic use of kettlebells in primary care is more likely to be informed by the fitness industry and practitioners in non-clinical roles, with the current body of evidence offering little guidance for this type of intervention. Applying the principles of mechanotherapy and a contemporary understanding of pain, kettlebells could be used therapeutically in the management of a wide range of common musculoskeletal conditions, although this remains to be demonstrated.

## Data Availability

The datasets used and/or analysed during the current study are available from the corresponding author on reasonable request.

## Abbreviations

1H: One handed swing
2H: Two-handed swing
ACL: Anterior Cruciate Ligament
ACLR: Anterior Cruciate Ligament Reconstruction
ACSM: American College of Sports Medicine
BLa: Blood Lactate
BP: Blood Pressure
ES: Effect Size
GRF: Ground Reaction Force
HR: Heart Rate
IKT: Incremental Kettlebell Swing Test
MET: Metabolic Equivalent
nEMG: Normalised Electromyograph
PPT: Pain Pressure Threshold
RCT: Randomised Controlled Trial
RER: Respiratory Exchange Rate
RKC: Russian Kettlebell Certification
RM: Repetition Maximum
RPE: Rate of Perceived Exertion
rpm: Revolutions Per Minute
SD: Standard Deviation
sEMG: Surface Electromyograph
SFG: Strong First Girya
SMD: Standardised Mean Difference
SPM: Swings Per Minute
ST: Semitendinosus
TGU: Turkish Get-up
VE: Minute Ventilation
VL: Vastus Lateralis
VO_2_: Oxygen consumption
